# Concerted Reactive Adsorption and Photocatalytic Degradation of Bisphenol‑S on Molybdenum Cluster-Modified Nanoceria

**DOI:** 10.1021/acs.inorgchem.5c02157

**Published:** 2025-09-03

**Authors:** Martin Št́astný, Jakub Tolasz, Dmytro Bavol, Matouš Kloda, Yoshiyuki Sugahara, Kamil Lang, Jiří Henych, Kaplan Kirakci

**Affiliations:** 1 112895 Institute of Inorganic Chemistry of the Czech Academy of Sciences, Husinec-Řež 250 68, Czech Republic; 2 Department of Applied Chemistry, Faculty of Science and Engineering, 13148 Waseda University, 3-4-1 Okubo, Shinjuku-ku, Tokyo 169-8555, Japan; 3 Kagami Memorial Institute for Materials Science and Technology, 13148 Waseda University, 2-8-26 Nishiwaseda, Shinjuku-ku, Tokyo 169-0051, Japan

## Abstract

Bisphenol-S (BPS) is a widespread toxic industrial pollutant and endocrine disruptor of growing environmental concern. This study investigates the use of porous nanoceria particles (CeO_2_) functionalized with octahedral molybdenum clusters (Mo_6_) for the removal of waterborne BPS through a combined mechanism of reactive adsorption and photodegradation. Although immobilization of Mo_6_ cluster reduced the overall surface area of CeO_2_, BPS adsorption and partial decomposition in the dark were enhanced due to chemical specificity and surface interactions introduced by the Mo_6_ clusters. Upon UV-A irradiation, quenching of the phosphorescence of the clusters indicated photoinduced electron transfer from Mo_6_ to CeO_2_, which facilitated hydroxyl radical generation and improved BPS photocatalytic degradation. Unlike bare nanoceria, the Mo_6_@CeO_2_ composite initially retained the intermediate 4-hydroxybenzenesulfonic acid formed in the dark, but subsequently released it, along with phenol and other degradation products, under light. This controlled photodesorption was coupled with stable performance over multiple degradation cycles. Under simulated solar irradiation, the composite achieved a 3-fold increase in BPS removal efficiency compared to bare nanoceria. These findings highlight the synergistic interplay between Mo_6_ clusters and nanoceria and reflects the potential of this composite material for effective water remediation.

## Graphical abstract

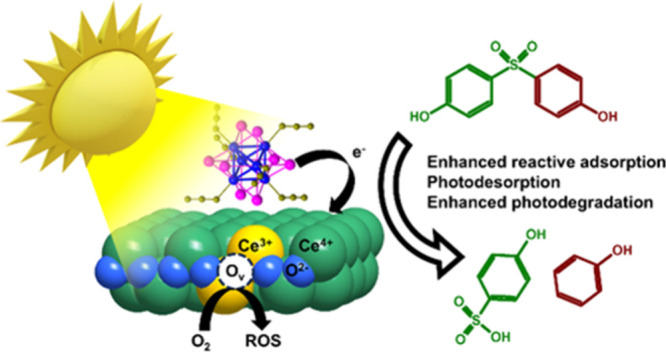

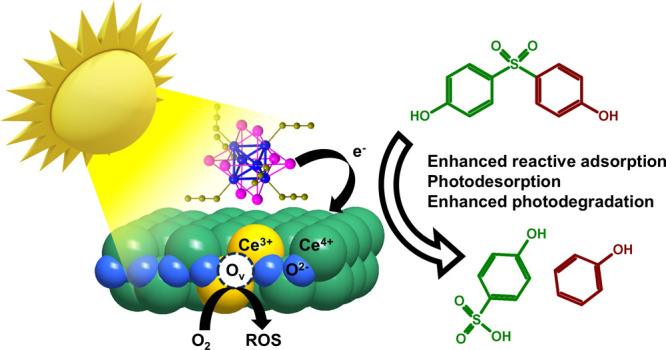

## Introduction

Water pollution poses a significant threat to human health and ecosystems globally. The photocatalytic degradation of harmful chemicals has come forth as a promising strategy for the removal of water pollutants. It is based on the production of reactive oxygen species (ROS) such as singlet oxygen, superoxide or hydroxyl radicals by materials exposed to ultraviolet or visible light irradiation. Such species are short-lived and the degradation products of the targeted pollutants are generally less toxic, making this approach safer than the use of chemical agents such as the widespread and controversial chlorine derivates.^1–3^ The endocrine disruptor bisphenol-S (BPS) is a contaminant of emerging concern and constitutes a good target for photocatalytic degradation. BPS is often used as a replacement of bisphenol A (BPA) due to its good thermal and light stability. However, BPS has higher solubility in water and can have similar toxic effects on humans and water organisms such as BPA.^4^ Moreover, its higher stability when compared to BPA, makes it more resistant to photocatalytic degradation.

Nanocrystalline cerium dioxide (i.e., nanoceria) is a well-known (photo)­catalyst with extraordinary redox and acid–base properties that give it unique surface properties and reactivity. This is relevant not only for C1 catalysis such as CO_2_ activation or water gas shift reaction,^5^ but also for reactive adsorption and hydrolytic cleavage of phosphate esters, e.g.,^6,7^ toxic pesticides, chemical warfare agents, but also biomolecules including DNA/RNA, the controlled dissociation of which is relevant for environmental and biological applications. Our recent results indicate that other compounds, such as sulfonamide drugs, can be also decomposed on nanoceria.^8^ Decomposition reactions can be further accelerated by irradiation with light, as ceria is semiconducting oxide, although its photocatalytic efficiency is relatively low due to limited absorption of visible light and high recombination rate of photogenerated charges.

Multicomponent systems or composites are attractive constructs for the design of photoactive materials for environmental applications as they allow for synergistic effects between their building blocks.^9^ One of the most efficient processes in this regard is the photoinduced electron transfer from the excited states of a dye to the conduction band of a semiconducting material, leading to the formation of ROS, mainly superoxide, hydrogen peroxide, or hydroxyl radicals via electron injection from the semiconductor to molecular oxygen. Systems displaying such process show enhanced photocatalytic or photoinactivation properties due to the extended visible-light absorption of the dye and the efficient electron/hole separation allowing for superior production of ROS.

Octahedral molybdenum clusters (Mo_6_), of the general formula [Mo_6_I_8_L_6_]^n^ (L = inorganic or organic apical ligand, −2 ≤ *n* ≤ 4, Figure 1) constitute efficient and sustainable components for the design of dye-sensitized photoactive systems. These red luminophores and singlet oxygen photosensitizers are readily excitable by light from the UV up to green spectral region to form long-lived excited triplet states.^10,11^ A wide range of possible apical ligands allows for tuning their physicochemical properties, such as absorption and phosphorescence properties, charge, aqueous stability, and hydrophobicity.^12–14^ In contrast to typical organic dyes such as porphyrins, the Mo_6_ clusters maintain their luminescent properties in the solid state and are less prone to photobleaching due to their metallic nature. These features enable the preparation of materials containing high concentrations of the photoactive Mo_6_ with efficient and robust luminescent properties.^15^ So far, the photodegradation of pollutants using Mo_6_-based materials has been investigated with nonrelevant targets such as rhodamine.^16,17^ Recently, semiconductor/Mo_6_ cluster composites have shown promising properties for bacterial photoinactivation,^18^ sensing,^19^ dye-sensitized solar-cell,^20^ or water-splitting.^21^

**Figure 1:**
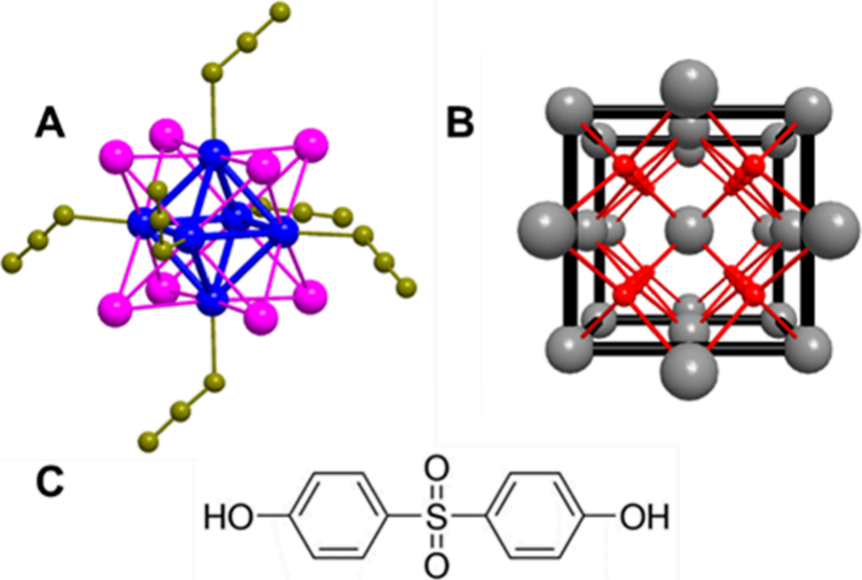
Schematic representations of the structures of [Mo_6_I_8_(N_3_)_6_]^2–^ (**Mo**_**6**_), color codes: Mo (blue), I (violet), N (dark yellow) (**A**), nanoceria (**CeO**_**2**_), color codes: Ce (gray), O (red) (**B**) and bisphenol-S (BPS) (**C**).

Herein we report on the photocatalytic degradation of BPS in aqueous media by a **Mo**_**6**_ **@CeO**_**2**_ composite material. The composite material was assembled in methanol by electrostatic interactions between nanoceria (**CeO**_**2**_), prepared by precipitation and refluxing in water without a high-pressure or high-temperature treatment, and cluster compound Na_2_[Mo_6_I_8_(N_3_)_6_ (**Mo**_**6**_) (Figure 1). All materials were characterized by transmission electron microscopy (TEM), dynamic light scattering (DLS), inductively coupled plasma mass spectrometry (ICP-MS), and Raman spectroscopy. Their photophysical properties evidenced photoinduced electron transfer from the Mo_6_ cluster to nanoceria, which leads to an enhanced photodegradation of BPS (Figure 1), when compared to bare nanoceria. In addition, the surface chemical properties of composites led to significant improvement of reactive adsorption of BPS, i.e., its initial degradation in the absence of light irradiation.

## Results and Discussion

### Syntheses and Characterization of **Mo**_**6**_ **@CeO**_**2**_ Composite

The **CeO**_**2**_ sample was prepared by precipitating an aqueous cerium­(III) nitrate solution with sodium hydroxide solution, followed by treatment with hydrogen peroxide and refluxing at 100 °C for 24 h, yielding 5 nm nanocrystals forming a porous secondary structure (Figure 2, Table 2).^8^ **Mo**_**6**_ **@CeO**_**2**_ composite was prepared by mixing nanoceria (**CeO**_**2**_) and Na_2_[Mo_6_I_8_(N_3_)_6_] (**Mo**_**6**_) in methanol for 3 days at room temperature. Unattached **Mo**_**6**_ were removed via several cycles of washing with acetone followed by centrifugation. TEM images of **Mo**_**6**_ **@CeO**_**2**_ documented aggregates of ∼ 5 nm ceria nanocrystals displaying their characteristic 0.32 nm interplanar distance (111) (Figure 2). EDS elemental mapping of **Mo**_**6**_ **@CeO**_**2**_ showed a homogeneous distribution of **Mo**_**6**_ within the porous structure formed by the aggregated nanoceria particles (Figure 2 and S1). ICP-MS elemental analysis evidenced a Mo:Ce mass ratio of 0.089, corresponding to a mass loading of **Mo**_**6**_ of approximately 24%, relative to nanoceria. Powder X-ray diffraction pattern of the composite closely resembled that of **CeO**_**2**_, indicating that immobilized **Mo**_**6**_ is amorphous and that the nanoceria crystal structure remained largely unchanged. Rietveld refinement confirmed the presence of **CeO**_**2**_ crystallites with an average size of approximately 5 nm (Figure S2, Table 1).

**Figure 2:**
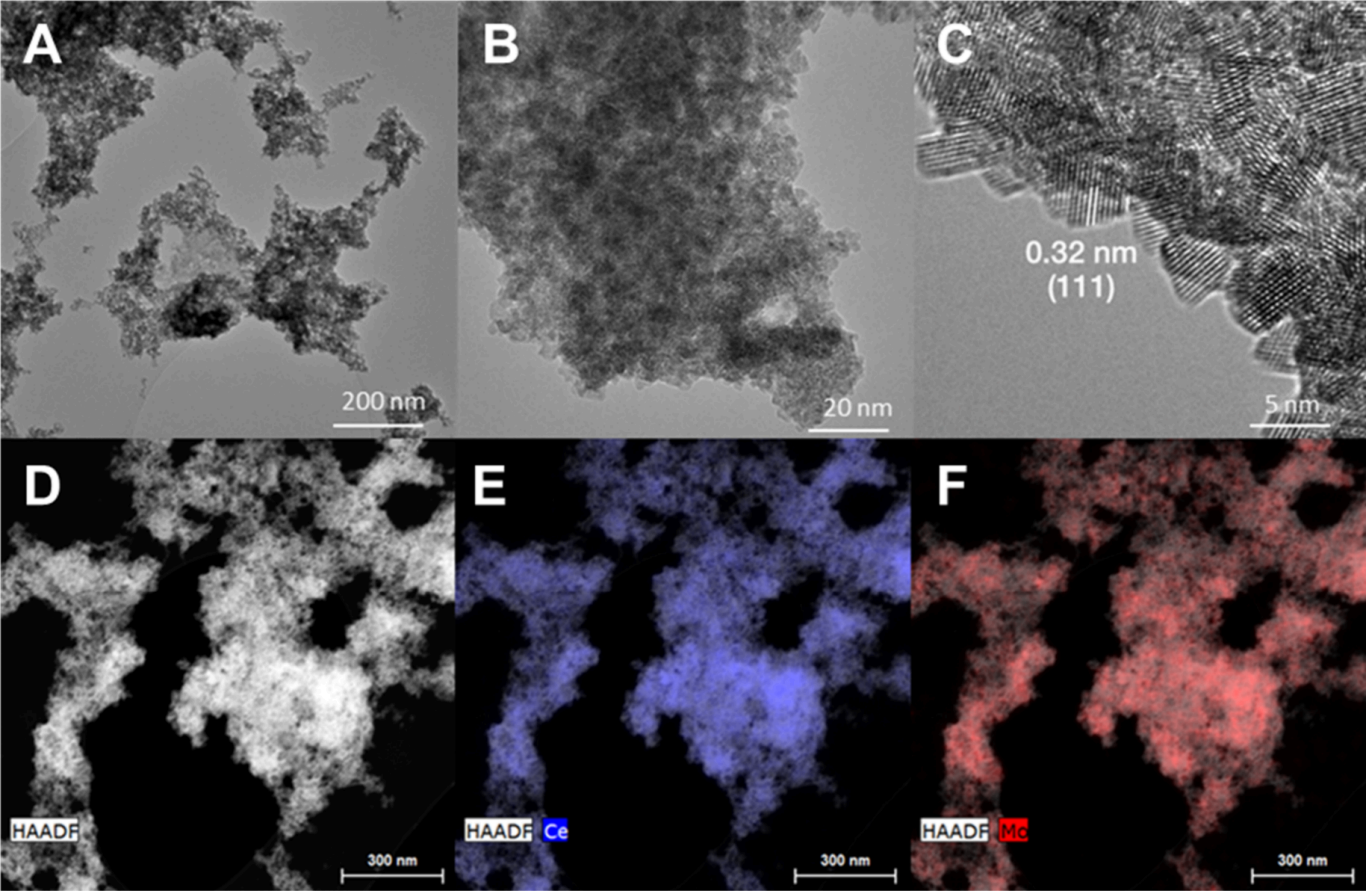
TEM images of **Mo**_**6**_ **@CeO**_**2**_ in the bright field (**A**, **B, C**), and in the dark field (**D**) with the corresponding EDS elemental mapping of Ce (**E**) and Mo (**F**). The interplanar distance of 0.32 nm in **2C** corresponds to (111) interplanar distance of nanoceria in **Mo**_**6**_ **@CeO**_**2**_.

**Table 1:** Structural Parameters of Nanoceria Obtained by the Rietveld Refinement

Sample	Crystallite size (XRD), nm	Lattice constant (*a*), Å	Microstrain (*ε*)
**CeO**_**2**_	5.1	5.4288	0.0088
**Mo**_**6**_ **@CeO**_**2**_	5.5	5.4260	0.0050

Gas sorption experiments showed that **CeO**_**2**_ displayed mesoporosity attributable to the voids formed by aggregation of primary nanoceria particles, with a BET surface of 189 m^2^ g^–1^. A decrease in BET surface area to 114 m^2^ g^–1^ after loading of **Mo**_**6**_ was observed for **Mo**_**6**_ **@CeO**_**2**_, in accordance with the efficient **Mo**_**6**_ loading evidenced with ICP-MS elemental quantification and TEM elemental mapping (Figure S3, Table 2).

**Table 2:** Sorption Properties of **CeO**_**2**_ and **Mo**_**6**_ **@CeO**_**2**_

Sample	BET surface area (m^2^ g^–1^)	Total pore volume at P/P_0_ = 0.99 (cm^3^ g^–1^)	Average pore diameter (4 V/A) (nm)
**CeO**_**2**_	189	0.19	3.9
**Mo**_**6**_ **@CeO**_**2**_	114	0.15	5.3

The Raman spectrum of **Mo**_**6**_ showed several well-defined vibrations in the low wavenumber region below 200 cm^–1^ and suppressed vibrations in the 200–350 cm^–1^ region, typical for Mo_6_ clusters and fairly consistent with previous reports (Figure 3A).^22^ The symmetric stretch of the azido ligands was also distinguishable at 1380 cm^–1^.^23^ The characteristic bands were observed in pure **CeO**_**2**_, i.e., the symmetric breathing vibration (F_2g_) at ∼ 455 cm^–1^ and Frenkel-type oxygen defects at ∼ 600 cm^–1^.^24^ The band at 1044 cm^–1^ corresponds to residual nitrates originating from the precursor, commonly observed in ceria synthesized by this method.^25^ In **Mo**_**6**_ **@CeO**_**2**_, the presence of the bands below 200 cm^–1^ and the bands at 263 and 307 cm^–1^, assignable to cluster′s vibration indicated the preservation of their molecular structure.^22^ The intensity of the symmetric stretch of the azido ligands was enhanced upon immobilization of the clusters into nanoceria and the band of residual nitrates at 1044 cm^–1^ disappeared. Nitrate anions were likely displaced by Mo_6_ clusters during the formation of **Mo**_**6**_ **@CeO**_**2**_ as negatively charged **Mo**_**6**_ (2-) have higher affinity toward nanoceria surface than NO_3_^–^. New bands also appeared at 865 and 940 cm^–1^ in the spectrum of the composite, possibly belonging to organic impurities. XPS spectra of **Mo**_**6**_, **CeO**_**2**_ and **Mo**_**6**_ **@CeO**_**2**_ ensured that the chemical integrity of the {Mo_6_I_8_} inorganic core was preserved within the composite (Figure 3B,C and S4). The signals of Mo 3d_3/2–5/2_ and I 3d_3/2–5/2_ appeared at the expected positions for Mo_6_ cluster, confirming that preservation of the inorganic core occurred during the preparation of the composite.^15^ Despite the presence of nitrogen in the composite as demonstrated by EDS, the XPS analysis of nitrogen was not possible for the composite due to too weak signals in the N 1s region, possibly because of strong X-ray absorption by the other heavy elements forming the composite. The Ce 3d core levels revealed Ce^3+^/Ce^4+^ ratios of 16.6 and 20.3%, for **CeO**_**2**_ and **Mo**_**6**_ **@CeO**_**2**_, respectively, suggesting that the presence of **Mo**_**6**_ stabilizes catalytically active Ce^3+^ centers in the composite (Figure 3D and S4).

**Figure 3:**
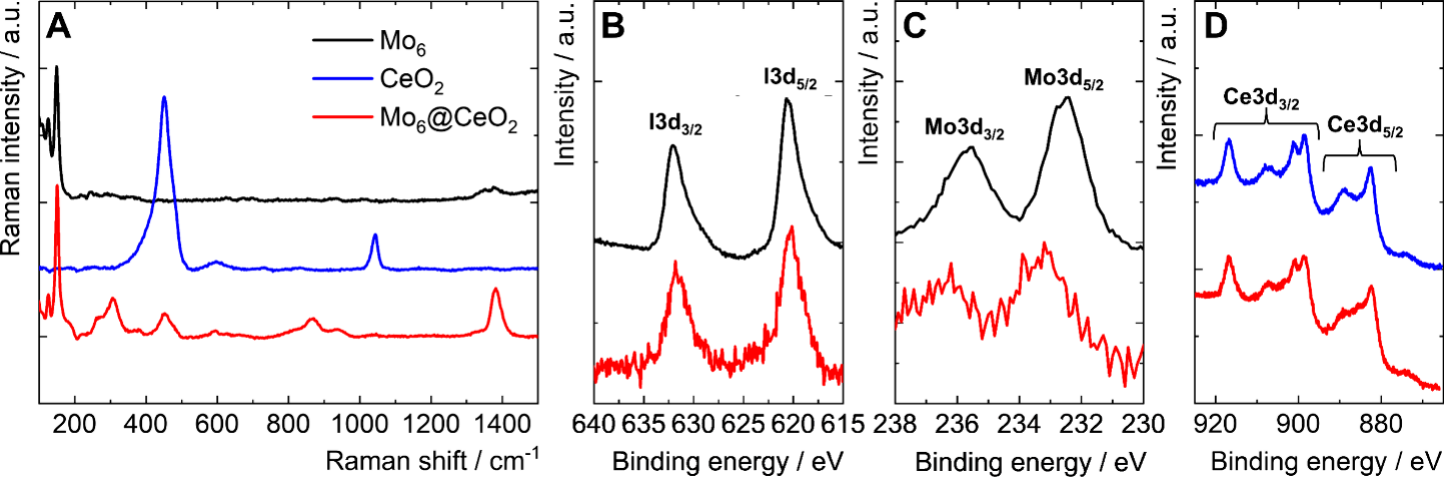
(**A**) Raman spectra of **Mo**_**6**_, **CeO**_**2**_, and **Mo**_**6**_ **@CeO**_**2**_. XPS spectra of the I 3d (**B**) and Mo 3d (**C**) core levels of **Mo**_**6**_ (black) and **Mo**_**6**_ **@CeO**_**2**_ (red). The Ce 3d signals of **CeO**_**2**_ (blue) and **Mo**_**6**_ **@CeO**_**2**_ (red) are compared in (D). All corresponding fits are given in Figure S4.

The colloidal parameters of a photocatalyst such as hydrodynamic diameter or surface charge can affect the efficiency of the photodegradation of waterborne pollutants. Thus, dynamic light scattering experiments were performed on dispersions of **Mo**_**6**_ **@CeO**_**2**_ and their individual components in deionized water (Figure S5, Table 3). **Mo**_**6**_ appeared as negatively charged nanoaggregates with a diameter of approximately 20 nm in deionized water with a zeta potential of −24 ± 17 mV, while **CeO**_**2**_ formed positively charged particles with a diameter of approximately 100 nm with a zeta potential of +30 ± 7 mV. Aqueous dispersion of **Mo**_**6**_ **@CeO**_**2**_ displayed slightly larger diameter than that of **CeO**_**2**_; surprisingly, the corresponding zeta potential of +29 ± 5 mV remained the same as that of bare nanoceria. This trend was preserved in methanol, with zeta potentials of −7 ± 16, + 14 ± 15, and +13 ± 17 mV for **Mo**_**6**_, **CeO**_**2**_, and **Mo**_**6**_ **@CeO**_**2**_, respectively. The unchanged zeta potential of **Mo**_**6**_ **@CeO**_**2**_, when compared to bare **CeO**_**2**_ in both solvents, can be attributed to the distribution of **Mo**_**6**_ clusters in the internal surface of the composite’s particles rather than at the external surface.

**Table 3:** Mean Size by Number, Z-Average Hydrodynamic Diameter, Polydispersity Index (PDI) and Zeta Potentials of Dispersions of the Composite Materials and Their Individual Components in Deionized Water at Room Temperature, as Obtained by Dynamic/electrophoretic Light Scattering

Sample	Mean size by number/nm	Z-average/nm	PDI	Zeta potential/mV
**Mo**_**6**_	19 ± 6	153	0.23	–24 ± 17 (−7 ± 16)^a^
**CeO**_**2**_	105 ± 43	157	0.21	+30 ± 7 (14 ± 15)^a^
**Mo**_**6**_ **@CeO**_**2**_	136 ± 56	200	0.18	+29 ± 5 (13 ± 17)^a^

a measured in methanol.

### Photophysical Studies

The extent of electronic interaction between the clusters and nanoceria was probed by studying the photophysical properties of aqueous dispersions of **Mo**_**6**_ **@CeO**_**2**_ and its individual components (Figure 4, Table 4). The absorption spectrum of **Mo**_**6**_ is typical for [Mo_6_I_8_L_6_]^2–^ clusters, with broad bands in the UV-blue region and on onset at approximately 550 nm. **CeO**_**2**_ displayed an absorption band in the UV-A region with maxima at approximately 325 nm. The absorption spectrum of **Mo**_**6**_ **@CeO**_**2**_ resemble a composite spectrum of **CeO**_**2**_ and **Mo**_**6**_. When excited at 400 nm, **Mo**_**6**_ showed typical broad phosphorescence band of [Mo_6_I_8_L_6_]^2–^ clusters with a maximum at 689 nm. The band of **Mo**_**6**_ was broadened and slightly red-shifted upon immobilization onto nanoceria. The absolute phosphorescence quantum yield of 0.24 for **Mo**_**6**_ dropped below the detection limit of our apparatus (<0.01) for **Mo**_**6**_ **@CeO**_**2**_ in argon-saturated aqueous dispersion. Similarly, the phosphorescence decay kinetics revealed a dramatic decrease in the phosphorescence lifetime from 104 μs for **Mo**_**6**_ to 1.2 μs for **Mo**_**6**_ **@CeO**_**2**_. Thus, **Mo**_**6**_ immobilized onto nanoceria experienced a strong quenching of their red phosphorescence, pointing to electronic interactions between **Mo**_**6**_ and nanoceria. Possibly, a fast photoinduced electron transfer from the excited states of **Mo**_**6**_ to nanoceria occurs in a similar fashion as we recently reported for **Mo**_**6**_ **@graphene oxide** composite.^18^ We hypothesize that the efficiency of the photoinduced electron transfer is promoted by the redox-active Ce^4+^/Ce^3+^ sites that chemically trap electrons, enhancing charge separation.^26^

**Figure 4:**
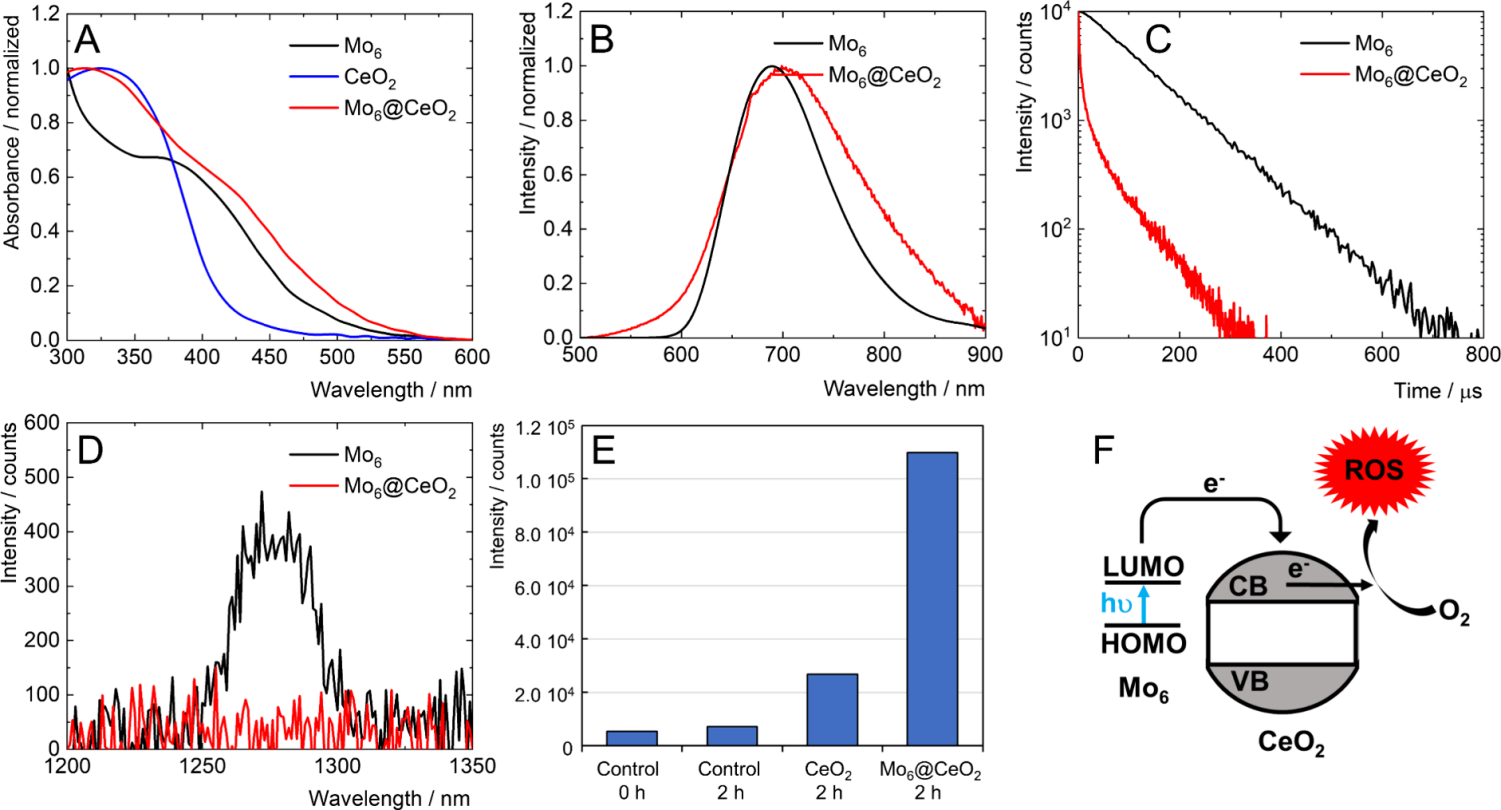
(**A**) Normalized absorption spectra of water dispersions of **Mo**_**6**_, **CeO**_**2**_, and **Mo**_**6**_ **@CeO**_**2**_. (**B**) Normalized emission spectra of Ar-saturated aqueous dispersions of **Mo**_**6**_ and **Mo**_**6**_ **@CeO**_**2**_ excited at 400 nm. (**C**) Phosphorescence decay kinetics of Ar-saturated aqueous dispersions of **Mo**_**6**_ and **Mo**_**6**_ **@CeO**_**2**_, excited at 400 nm and recorded at the phosphorescence maximum. (**D**) Phosphorescence signal of O_2_(^1^Δ_g_) in air-saturated aqueous dispersions of **Mo**_**6**_ and **Mo**_**6**_ **@CeO**_**2**_, excited at 400 nm. (**E**) Fluorescence intensity of 7-hydroxycoumarin measured at 460 nm after 2 h of irradiation of **CeO**_**2**_ and **Mo**_**6**_ **@CeO**_**2**_ water dispersions (1 mg mL^–1^) containing 50 μM coumarin. The dispersions were irradiated with a blue LED light source. 50 μM coumarin water solutions in the dark and after irradiation were used as controls. (**F**) Schematic representation of electronic states involved in the photoinduced electron transfer from **Mo**_**6**_ to **CeO**_**2**_ and the resulting ROS formation.

**Table 4:** Photophysical Properties of **Mo**_**6**_ and **Mo**_**6**_ **@CeO**_**2**_ in Deionized Water at Room Temperature^a^

Sample	*λ*_*L*_/nm	*Φ*_*L*_	*τ*_*L*_/μs	*τ*_*air*_/μs
**Mo**_**6**_	689	0.26	104	5.0
**Mo**_**6**_ **@CeO**_**2**_	702	<0.01	1.2	1.0

a *λ*_*L*_ - phosphorescence maximum (λ_exc_ = 400 nm); *τ*_*L*_ and *τ*_*air*_ - amplitude average lifetimes in Ar and air-saturated deionized water, recorded at the maximum of emission (λ_exc_ = 400 nm); *Φ*_*L*_ phosphorescence quantum yield (λ_exc_ = 400 nm, experimental error of *Φ*_*L*_ is ± 0.01).

A drop in the amplitude average lifetime from 104 μs in oxygen-free aqueous solution to 5.0 μs in air-saturated aqueous solution for **Mo**_**6**_ dispersions indicates strong phosphorescence quenching by oxygen, typical for **Mo**_**6**_ complexes in solutions (Figure S6).^11^ This feature was lost for **Mo**_**6**_ **@CeO**_**2**_ dispersions, with only minute change in the lifetimes between oxygen-free and air-saturated aqueous dispersions. Such behavior suggests a poor singlet oxygen photosensitizing ability for the composite, as confirmed by measuring the phosphorescence signal of O_2_(^1^Δ_g_) centered at 1274 nm which was clearly detectable for air-saturated water solution of **Mo**_**6**_, but absent for **Mo**_**6**_ **@CeO**_**2**_ (Figure 4D). We also investigated the generation of hydroxyl radicals, a key ROS involved in photocatalytic processes. If photoinduced electron transfer occurs as hypothesized, irradiation with blue light should results in increased hydroxyl radical production. To assess this, we employed the coumarin assay, wherein coumarin reacts with hydroxyl radicals to form the highly fluorescent product 7-hydroxycoumarin. Upon irradiation with blue LED light for 2 h, significant increases in fluorescence intensity of 7-hydroxycoumarin were observed for both **CeO**_**2**_ and **Mo**_**6**_ **@CeO**_**2**_ dispersions containing 50 μM of coumarin (Figure 4E). This fluorescence enhancement clearly indicates hydroxyl radical formation. Notably, **Mo**_**6**_ **@CeO**_**2**_ exhibited more than a 5-fold increase in hydroxyl radical production compared to **CeO**_**2**_ alone**_._** These findings suggest that in the composite, the typical singlet oxygen photosensitization pathway of **Mo**_**6**_ is bypassed by fast photoinduced electron transfer from its excited triplet states to the conduction band of nanoceria, thereby enhancing hydroxyl radical generation via the nanoceria matrix (Figure 4F).

### Adsorption and UV-A Photodegradation of BPS

BPS was used as a model compound to evaluate the adsorption and UV-A photodegradation performance of the composite and its individual components in aqueous solution. A detailed investigation of BPS degradation kinetics was conducted using high-performance liquid chromatography with diode-array detection (HPLC-DAD). The kinetic profiles shown in Figure 5, along with the corresponding parameters calculated using the pseudo-first-order kinetic model (Equation S1, Table S1), highlighted significant differences in adsorption and degradation efficiencies between the composite and its individual components. We uncovered a complex interplay between reactive adsorption (i.e., spontaneous degradation of BPS at the surface of material in the dark), photocatalytic degradation (i.e., degradation of BPS by the material upon light irradiation), and photodesorption processes (i.e., photoinduced release of adsorbed degradation products into solution).

**Figure 5:**
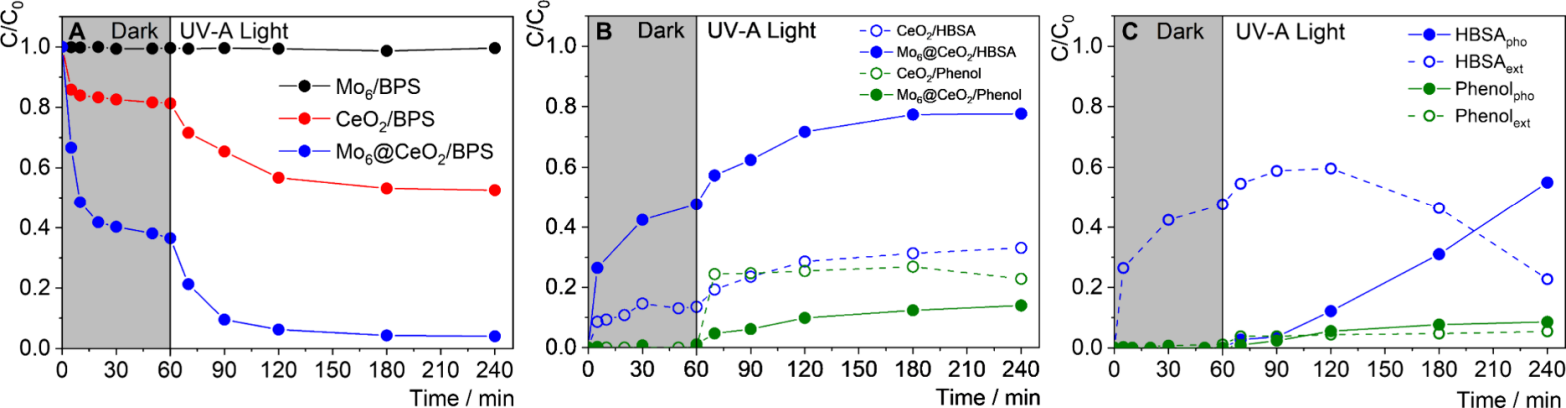
(**A**) Kinetic curves of adsorption (in the dark, first 60 min) and UV-A photocatalytic degradation of BPS (10 mg mL^–1^) on **Mo**_**6**_ (2.5 mg mL^–1^), **CeO**_**2**_ (2.5 mg mL^–1^) and **Mo**_**6**_ **@CeO**_**2**_ (2.5 mg mL^–1^). (**B**) Total concentrations of HBSA and phenol on **CeO**_**2**_ (no extraction required) and **Mo**_**6**_ **@CeO**_**2**_ (i.e., sum of photodesorbed and extracted concentrations). (**C**) Formation of major degradation products HBSA and phenol on **Mo**_**6**_ **@CeO**_**2**_, separated into photodesorbed (*pho*) and extracted (*ext*) concentrations. Extraction was performed using an acetonitrile:H_2_O (1:1, v/v) mixture.

Adsorption experiments conducted in the dark revealed that immobilizing **Mo**_**6**_ onto nanoceria greatly enhanced the BPS adsorption capacity D_dark_ from approximately 19% for **CeO**_**2**_ to 62% for **Mo**_**6**_ **@CeO**_**2**_, albeit with a slower rate (Figure 5A, Table S1). Thus, the BPS adsorption capacity of **Mo**_**6**_ **@CeO**_**2**_ composite surpasses that of bare **CeO**_**2**_, despite a reduction in the specific surface area. As illustrated in Figure 5A, **Mo**_**6**_ alone exhibited neither adsorption nor catalytic activity in the dark, indicating that its primary role in composite is to enhance adsorption rather than to directly participate in the catalytic process. The enhanced BPS adsorption is primarily attributed to the chemical specificity and surface interactions introduced by **Mo**_**6**_. **Mo**_**6**_ create high-affinity binding sites, enabling composite to absorb BPS more effectively despite its lower BET surface area. Upon UV-A irradiation, the photocatalytic degradation rate of BPS on **Mo**_**6**_ **@CeO**_**2**_ (k_UV_ ∼ 0.063 min^–1^) was approximately twice that of **CeO**_**2**_ alone (k_UV_ ∼ 0.030 min^–1^), while **Mo**_**6**_ itself showed no photocatalytic activity. Consequently, after 60 min in the dark and 180 min of UV-A exposure, **Mo**_**6**_ **@CeO**_**2**_ removed nearly all BPS from the solution, whereas approximately 50% of the initial BPS concentration remained when using **CeO**_**2**_ under identical conditions (Figure 5A).

Distinct differences were observed in the release mechanisms of degradation intermediates. In the dark, **CeO**_**2**_ primary released 4-hydroxybenzenesulfonic acid (HBSA), a major degradation product. In contrast, **Mo**_**6**_ **@CeO**_**2**_ exhibited a strong interaction with HBSA, leading to its retention on the surface and requiring extraction for accurate quantification (Figure 5B, C). Under UV-A irradiation, photodesorption of HBSA from **Mo**_**6**_ **@CeO**_**2**_ occurred, along with the release of another primary key intermediate, phenol. Notably, the amount of phenol released and extracted from **Mo**_**6**_ **@CeO**_**2**_ was lower than expected based on its stoichiometric correspondence with HBSA observed for bare **CeO**_**2**_. This discrepancy prompted further investigation, revealing that phenol undergoes additional photodegradation on the **Mo**_**6**_ **@CeO**_**2**_ surface, accounting for the observed imbalance. As shown in Figure S7, **Mo**_**6**_ **@CeO**_**2**_ exhibited minimal phenol adsorption in the dark, but demonstrated significant UV-A-induced photocatalytic degradation of phenol.

### LC-HRMS Analysis and Proposed Degradation Mechanism

High-performance liquid chromatography coupled with high-resolution mass spectrometry (LC-HRMS) allowed us to perform an inventory of the degradation products resulting from both reactive adsorption and UV-A photocatalytic degradation of BPS on **Mo**_**6**_ **@CeO**_**2**_. The initial BPS standard was characterized by the retention time (t_R_ = 4.82 min) and corresponding HRMS spectrum (Figures S8 and S9). Time-resolved chromatograms, displayed in Figure S10, provided a detailed view of BPS degradation, evidenced by the reduction of the BPS peak (peak 5, t_R_ = 4.82 min) and the emergence of degradation product peaks.

HBSA (peak 1, t_R_ = 1.79 min) was identified as a primary degradation product, appearing under UV-A irradiation. Interestingly, two distinct peaks (1 and 1’) with the identical mass spectra (Figures S12 and S13) were identified, suggesting the presence of two isomers of HBSA. Thus, the appearance of the peak 1’ (t_R_ = 2.01 min) under UV-A exposure points to a photochemical isomerization of HBSA. Figure S11, which shows the chromatogram of extracted products, documented that HBSA is also formed in the dark phase. It indicates reactive adsorption of BPS onto the composite surface and its catalytic degradation.

Further analysis of the chromatograms (Figure S10) showed the emergence of further degradation products under UV-A irradiation. Phenol (peak 4, t_R_ = 4.57 min), with its corresponding mass spectrum in Figure S14, was among these. Peaks 6 and 7 (t_R_ = 5.14 and 7.87 min, respectively) exhibited mass spectra identical to the BPS standard (Figure S15) and were attributed to BPS isomers (BPS iso1 and BPS iso2), possibly 2,4’-dihydroxydiphenyl sulfone and 2,2’-dihydroxydiphenyl sulfone, suggesting ROS-mediated conformational or structural rearrangement (Figure 6).^27^ Furthermore, the peak 2 (t_R_ = 3.10 min), with a parent ion at *m*/*z* 328.9795 (Figure S16), and the peak 3 (t_R_ = 3.58 min), with a parent ion at *m*/*z* 252.9482 (Figure S17), detailed in the zoomed-in area of Figure S10, were identified as sulfonic acid derivatives, respectively, suggesting the addition of the SO_3_H group to phenol and BPS. Note that the peak 2 is observable also in the BPS standard as a minor impurity, but its proportion increased several times during the photocatalytic phase of the experiment. The time-resolved distribution of BPS and degradation products released to solution are comprehensively presented in Figure S18, while a tentative degradation mechanism, including all the identified intermediates, is illustrated in Figure 6.

**Figure 6:**
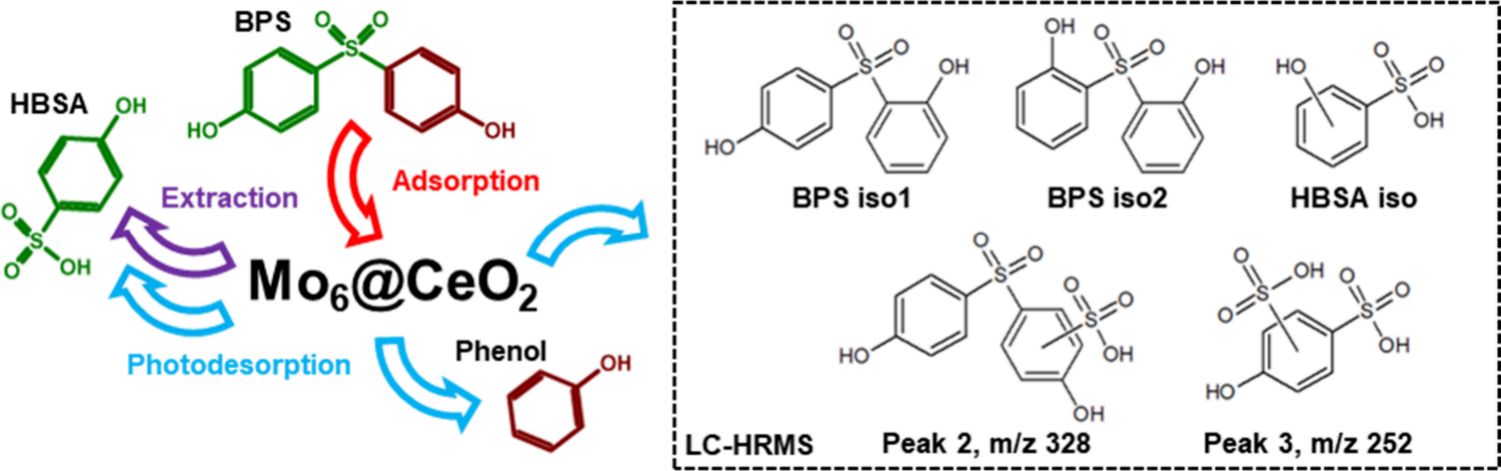
Proposed degradation scheme of BPS on **Mo**_**6**_ **@CeO**_**2**_ composite.

### Recyclability of Composite Photocatalyst

Given the high degradation efficacy of **Mo**_**6**_ **@CeO**_**2**_ for BPS, its recyclability was also evaluated. Figure 7A illustrates the recycling performance of the **Mo**_**6**_ **@CeO**_**2**_ composite across multiple cycles of adsorption and photocatalytic degradation. The results show that the composite retains a high degradation capacity for at least through four consecutive cycles, demonstrating its stability and durability. Specifically, the degradation efficiency was 99% in the first cycle and decreases to approximately 88% by the fourth cycle - a gradual decline is likely due to minor material losses during handling. The demonstrated reusability of **Mo**_**6**_ **@CeO**_**2**_ underscores its potential as a sustainable material for water treatment applications, enabling reduced material consumption and lower long-term operational costs.

**Figure 7:**
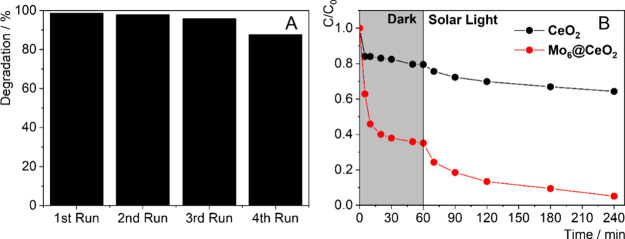
(**A**) Recycling performance of **Mo**_**6**_ **@CeO**_**2**_ during BPS degradation in four reuse cycles. (**B**) Kinetic curves of PBS adsorption (in the dark) and photocatalytic degradation under solar-simulated light on **CeO**_**2**_ and **Mo**_**6**_ **@CeO**_**2**_.

### Simulated Solar Light Photodegradation of PBS

The **Mo**_**6**_ **@CeO**_**2**_ composite was also evaluated for its photocatalytic performance under solar light irradiation for the degradation of BPS, as shown in Figure 7B and summarized in Table S1. While **CeO**_**2**_ achieved moderate PBS removal, reaching approximately 36%, **Mo**_**6**_ **@CeO**_**2**_ exhibited significantly enhanced activity, achieving nearly 95% removal under the same conditions. This substantial improvement indicates a higher photocatalytic degradation rate compared to bare **CeO**_**2**_. The enhanced efficiency of the composite under solar light can be attributed to the broader visible-light absorption introduced by the integration of **Mo**_**6**_ with **CeO**_**2**_. In the **Mo**_**6**_ **@CeO**_**2**_ composite, **Mo**_**6**_ extends the light-harvesting range and transfer photoexcited electrons to **CeO**_**2**_′s conduction band. This electron transfer promotes the formation of ROS, which play a crucial role in the degradation of BPS. As a result, **Mo**_**6**_ **@CeO**_**2**_ can efficiently harness natural sunlight, making it a promising material for practical environmental remediation applications.

## Conclusion

This study presents the preparation and characterization of Mo_6_-cluster-functionalized nanoceria composites, **Mo**_**6**_ **@CeO**_**2**_, and evaluates their effectiveness in adsorptive and photocatalytic degradation of BPS in aqueous environments. Photophysical studies confirmed the strong electronic interaction between **Mo**_**6**_ and nanoceria, leading to significant quenching of **Mo**_**6**_ phosphorescence, indicative of efficient photoinduced electron transfer. This interaction appears to be crucial for the enhancement of photocatalytic degradation by **Mo**_**6**_ **@CeO**_**2**_, which exhibits nearly complete removal of BPS under UV-A irradiation. The attachment of **Mo**_**6**_ onto nanoceria extends light absorption into the visible region and enhances the generation of ROS, thereby accelerating the degradation process. Notably, the synergetic interplay between **Mo**_**6**_ and **CeO**_**2**_ significantly improves reactive adsorption of BPS, initiating its efficient removal from water and partial degradation even in the absence of light. Finally, recyclability tests underscore the composite’s potential as a sustainable material for water treatment applications, demonstrating consistent degradation efficiency across multiple cycles. Its ability to effectively harness solar light for pollutant degradation further highlights its appeal for real-world environmental remediation applications. The **Mo**_**6**_ **@CeO**_**2**_ composite, in particular, stands out due to its superior structural properties and photocatalytic efficiency, making it a promising candidate for the degradation of organic pollutants under both artificial and natural light conditions. This work paves the way for further exploration of nanoceria-based composites in photocatalysis, with potential implications for cleaner and more sustainable environmental technologies.

## Experimental Section

### Reagents and General Procedures

Compound Na_2_[Mo_6_I_8_(N_3_)_6_] and nanoceria were prepared according to previously published procedures.^28,8^ Molybdenum, iodine, sodium azide, cerium­(III) nitrate hexahydrate, sodium hydroxide, and hydrogen peroxide were obtained from Sigma-Aldrich and used as received. Solvents for synthesis were purchased from Penta (Czech Republic) and dried over molecular sieves (3 Å).

Raman spectra were recorded with a DXR Raman confocal microscope (Thermo Fisher Scientific) using a 532 nm excitation laser. X-ray photoelectron spectroscopy (XPS) measurements were performed using a JEOL JPS-9010TR apparatus (source Mg Kα 10 kV, 10 mA). Images of the nanoparticles were acquired by a JEOL-2100 transmission electron microscope (JEOL, Japan). Size distributions and corresponding zeta potentials were determined by dynamic light scattering (DLS) on a particle size analyzer Zetasizer Nano ZS (Malvern, UK). Powder X-ray diffraction (XRD) patterns were recorded using a PANalytical X’Pert PRO diffractometer in the transmission setup equipped with a conventional Cu X-ray tube (40 kV, 30 mA). The molybdenum and cerium content of the composite was measured by inductively coupled plasma mass spectrometry (ICP-MS, PerkinElmer, Concord, ON, Canada). The sample was isolated by centrifugation (10 000 rpm, 5 min), and drying of the solid under reduced pressure for 24 h. Quantification was carried out via external calibration. The ICP-MS measurement conditions were as follows: RF power 1.1 kW, nebulizer gas flow rate 0.76 L min^–1^, auxiliary gas flow rate 1 L min^–1^, plasma gas flow rate 11 L min^–1^, measured isotope ^98^ Mo as an analyte and ^115^In as an internal standard. Luminescence properties were measured on an FLS1000 spectrometer using a cooled PMT-900 photon detection module (Edinburgh Instruments, UK). Dispersions in deionized water (0.1 mg mL^–1^) were saturated with air or argon to ensure different oxygen concentrations for phosphorescence analyses. The FLS1000 spectrometer was also used for time-resolved phosphorescence measurements (λ_exc_ = 405 nm, VPLED Series) and the recorded decay curves were fitted to exponential functions by the Fluoracle software (v. 2.13.2, Edinburgh Instruments, UK). Phosphorescence quantum yields and absorption spectra of the samples were recorded using a Quantaurus QY C11347–1 spectrometer equipped with an integration sphere (Hamamatsu, Japan). Singlet oxygen phosphorescence was measured on a Fluorolog 3 spectrometer using an H10330C-75-C3 photomultiplier (Hamamatsu Photonics, Japan). In this case, aqueous dispersions of similar absorbance were saturated with oxygen to magnify phosphorescence signals of O_2_(^1^Δ_g_). Hydroxyl radical generation was detected via its reaction with coumarin, which produces fluorescent 7-hydroxycoumarim. Dispersions of the photocatalysts (1 mg mL^–1^) in deionized water containing 50 μM coumarin were irradiated 2 h with a 460 nm LED lamp (Cameo studio PAR 6 G2) and the photocatalysts were removed by centrifugation (10 000 rpm/5 min). Fluorescence intensity of the solutions was measured at 460 nm (excitation at 343 nm). Deionized water with 50 μM coumarin served as a control.

### Preparation of Mo_6_@CeO_2_

A methanol solution of Na_2_[Mo_6_I_8_(N_3_)_6_] (100 mg, 10 mL) was added to a methanol dispersion of nanoceria (100 mg **CeO**_**2**_ in 10 mL of methanol). The mixture was left under magnetic stirring for 3 days. Then, the mixture was submitted to three cycles of centrifugation (10 000 rpm/10 min) and redispersion in 20 mL of acetone and in order to remove unattached cluster complexes. Fresh aqueous dispersions of the composite were prepared by centrifugation of the acetone dispersions and redispersion in deionized water. **Caution!** *Na*_*2*_ *[Mo*_*6*_ *I*_*8*_ *(N*_*3*_ *)*_*6*_ *] is potentially shock and temperature sensitive and should be handled with care on an appropriate scale, using personal protection precautions.*

### Adsorption, Photocatalytic Activity, and Recyclability

The experimental evaluation of the activity was conducted by assessing their efficacy in the degradation of the model compound, BPS. The initial concentration of BPS was 10 mg L^–1^. The experimental setup and procedures are outlined as follows: dispersions of nanoceria, **Mo**_**6**_, or the composite at a concentration of 2.5 mg mL^–1^ were prepared by dispersing the material in 50 mL of deionized water in a glass crystallization dish. This dispersion underwent ultrasonic treatment in an ultrasonic bath for 10 min to achieve homogeneous mixing and dispersion of the material. The resulting dispersion was then transferred to a custom-built photoreactor, which consisted of a closable benchtop box equipped with a laboratory lifting platform and overhead lighting. The lighting system comprised three air-cooled, conventional UV lamps (PL-S 230 V/11 W), emitting UV-A radiation with a peak wavelength of 365 nm and an irradiance of 3.25 mW cm^–2^ at the surface of the dispersion. The dispersion was stirred in the dark for 60 min to facilitate the preadsorption of BPS onto the catalyst surface. Following the dark phase, the UV-A lamps were activated, and the mixture was irradiated under stirring for the next 240 min. During both the dark and irradiation phases, 1 mL aliquots of the reaction mixture were periodically sampled, centrifuged at 18,000 rpm, and the supernatant was immediately analyzed using HPLC-DAD.

**Mo**_**6**_ **@CeO**_**2**_ was also evaluated for recyclability over multiple degradation cycles. After each cycle, the reaction mixture (50 mL) was centrifuged at 5,000 rpm for 5 min. The recovered solid material was redispersed in deionized water and centrifuged three times to maintain a consistent catalyst concentration.

For solar-light simulation experiments, a scaled-down setup was employed. A LS0400 solar simulator (Quantum Design USA) was equipped with a fiber optic coupling including lamp housing, power supply, cables, and adapters. The distance between the light source and a quartz cuvette edge was maintained at 75 mm. The quartz cuvette had a total volume of 3.5 mL and the reaction mixture was scaled proportionally: 700 μL of dispersion (2.5 mg mL^–1^) was mixed with 2450 μL of water and 350 μL of BPS solution (100 mg L^–1^), resulting in a final BPS concentration of 10 mg L^–1^.

Extraction tests were performed using an extraction solvent composed of a 1:1 mixture of acetonitrile and water for selected times with the entire catalyst load, carried out three times consecutively. In the first step, the supernatant was separated from the mixture by centrifugation (10,000 rpm for 10 min), followed by the addition of 5 mL of extraction solvent, in which the material was dispersed using a vortex mixer. The extract was again separated and transferred to a 25 mL volumetric flask. This extraction procedure was repeated twice, and the final volume was adjusted to the mark with the extraction solvent. The prepared solution was immediately analyzed by HPLC-DAD.

### HPLC-DAD and LC-HRMS Analysis of Degradation Products

The separation and identification of degradation products was conducted on a Vanquish Core HPLC system equipped with a diode array detector (DAD) and connected to an Orbitrap Exploris 120 mass spectrometer. The chromatographic conditions were as follows: Accucore PFP column (2.6 μm, 150 × 4.6 mm I.D.) maintained at 30 °C; gradient elution with acetonitrile (ACN)/water (0.1% formic acid, HPLC gradient grade, Aldrich) from 30/70 (0 min) to 95/5 (11 min), held at 95/5 (1 min), then returning linearly to 30/70 (in 30 s) for equilibration (2.5 min). The flow rate was set to 0.7 mL min^–1^, and detection was performed using the DAD in the range of 190–800 nm. Standards were dissolved in water to a final concentration of 10.0 mg L^–1^. The injection volume for all samples was 20 μL.

High-resolution mass spectrometry (HRMS) measurements were performed using a heated electrospray ionization (HESI) probe in the negative mode with nitrogen (4.8, Air Products) as a collision gas. The isotopic distribution observed in the mass spectra was fully consistent with the calculated spectral pattern. The ESI interface conditions were as follows: vaporizer temperature set to 60 °C; nitrogen as a nebulizing sheath gas and auxiliary gas, with flow rates of 60 arb. and 15 arb., respectively; spray voltage of 3 kV; ion transfer tube temperature of 320 °C; RF lens at 20%; and a mass range from 50 to 600.

Routine analyses were performed using an UltiMate 3000 HPLC system, with the same column and mobile phase composition as used in the LC-HRMS analysis.

## Supplementary Material



## Notes

TEM images, powder X-ray diffraction patterns, adsorption isotherms, XPS spectra, DLS data, phosphorescence data, kinetic parameters, chromatogram, HRMS spectra (PDF)
