# Supplementary material for: Concerted Reactive Adsorption and Photocatalytic Degradation of Bisphenol‑S on Molybdenum Cluster-Modified Nanoceria
Source: Inorg Chem. 2025 Sep 3;64(36):18166–74. doi: 10.1021/acs.inorgchem.5c02157 (PMC12442080; doi:10.1021/acs.inorgchem.5c02157)

Supporting Information

Concerted Reactive Adsorption and Photocatalytic Degradation of Bisphenol-S on Molybdenum Clusters-Modified Nanoceria

Martin Šťastný[1], Jakub Tolasz[1], Dmytro Bavol[1], Matouš Kloda[1], Yoshiyuki Sugahara[2,3], Kamil Lang[1], Jiří Henych[1*], Kaplan Kirakci[1*]

[1]Institute of Inorganic Chemistry of the Czech Academy of Sciences, 250 68 Husinec-Řež, Czech Republic

[2]Department of Applied Chemistry, Waseda University, Faculty of Science and Engineering, 3-4-1 Okubo, Shinjuku-ku, Tokyo 169-8555, Japan

[3]Kagami Memorial Institute for Materials Science and Technology, Waseda University, 2-8-26 Nishiwaseda, Shinjuku-ku, Tokyo 169-0051, Japan

Corresponding authors' email addresses: Kaplan Kirakci kaplan@iic.cas.cz, Jiří Henych henych@iic.cas.cz

Content

Figure S1. TEM images of $\mathbf{Mo_6@CeO_2}$ in in the dark field with the corresponding EDS elemental mapping of Ce, Mo, I, and N.

Figure S2. Powder X-ray diffraction patterns with the Rietveld fit.

Figure S3. Adsorption isotherms.

Figure S4. XPS spectra of the Mo3d, I3d and Ce3d core levels of $\mathbf{Mo_6}$, $\mathbf{CeO_2}$ and $\mathbf{Mo_6@CeO_2}$ with corresponding fits.

Figure S5. Size distribution by number and zeta potential distribution in deionized water (pH~6), as obtained by dynamic/electrophoretic light scattering.

Figure S6. Phosphorescence decay kinetics of Ar- and air-saturated aqueous dispersions.

Kinetic analysis. Kinetic equation expressing change in concentration of BPS over time.

Table S1. Kinetic parameters of BPS adsorption in the dark and photocatalytic degradation under UV-A and solar light irradiation on $\mathbf{CeO_2}$ and $\mathbf{Mo_6@CeO_2}$.

Figure S7. Kinetic curves of adsorption (in the dark) and UV-A photocatalytic degradation of phenol on $\mathbf{Mo_6@CeO_2}$

Figure S8. Chromatograms of the blank and standard of BPS.

Figure S9. HRMS spectra of the BPS standard.

Figure S10. Time-resolved chromatograms of the BPS adsorption and photodegradation on $\mathbf{Mo_6@CeO_2}$.

Figure S11. Time-resolved chromatograms of extracts after BPS adsorption and photodegradation on $\mathbf{Mo_6@CeO_2}$.

Figure S12. HRMS spectra of the HBSA.

Figure S13. HRMS spectra of the HBSA isomer formed during photocatalytic degradation of BPS.

Figure S14. HRMS spectra of the peak 4 (phenol).

Figure S15. HRMS spectra of BPS and its isomers.

Figure S16. HRMS spectra of the peak 2.

Figure S17. HRMS spectra of the peak 3.

Figure S18. Time distribution of all products released to solution, during UV-A photocatalytic degradation of BPS on $\mathbf{Mo_6@CeO_2}$, expressed as the peak area from LC-HRMS analyses.

Figure S1. TEM images of **Mo_6@CeO_2** in in the dark field with the corresponding EDS elemental mapping of Ce, Mo, I, and N.

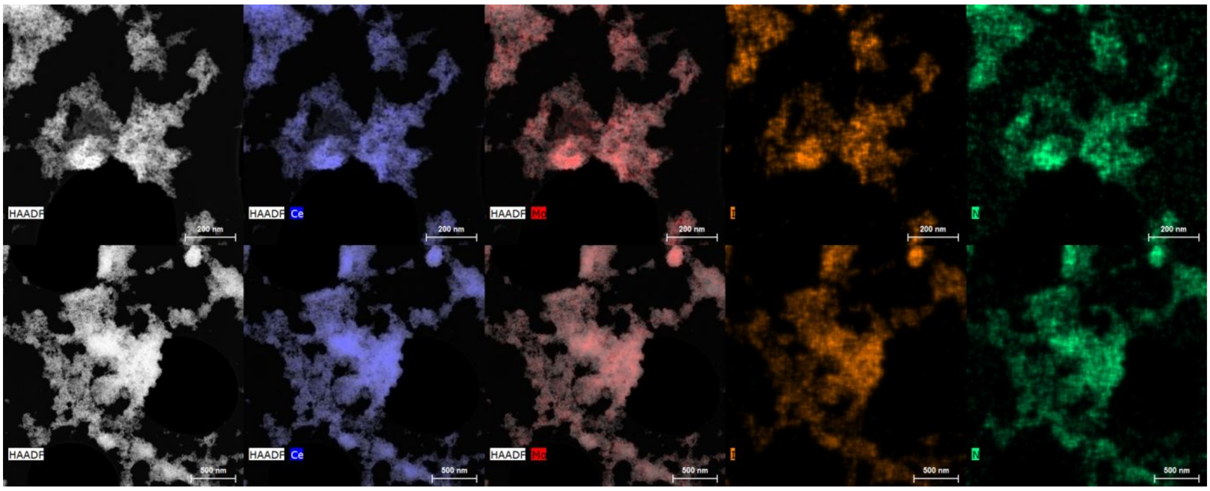

Figure S2. Powder X-ray diffraction patterns with the Rietveld fit of **CeO_2** and **Mo_6@CeO_2**.

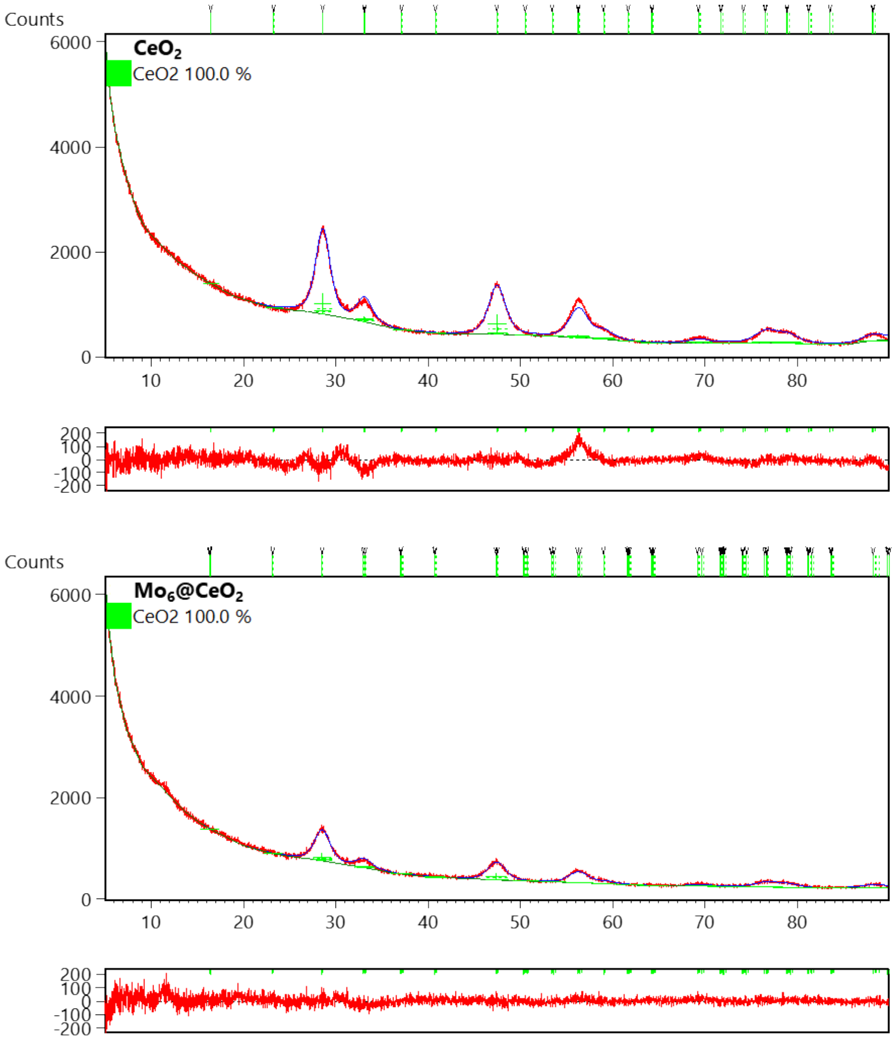

Figure S3. Adsorption isotherms of $\mathbf{CeO_2}$ and $\mathbf{Mo_6@CeO_2}$.

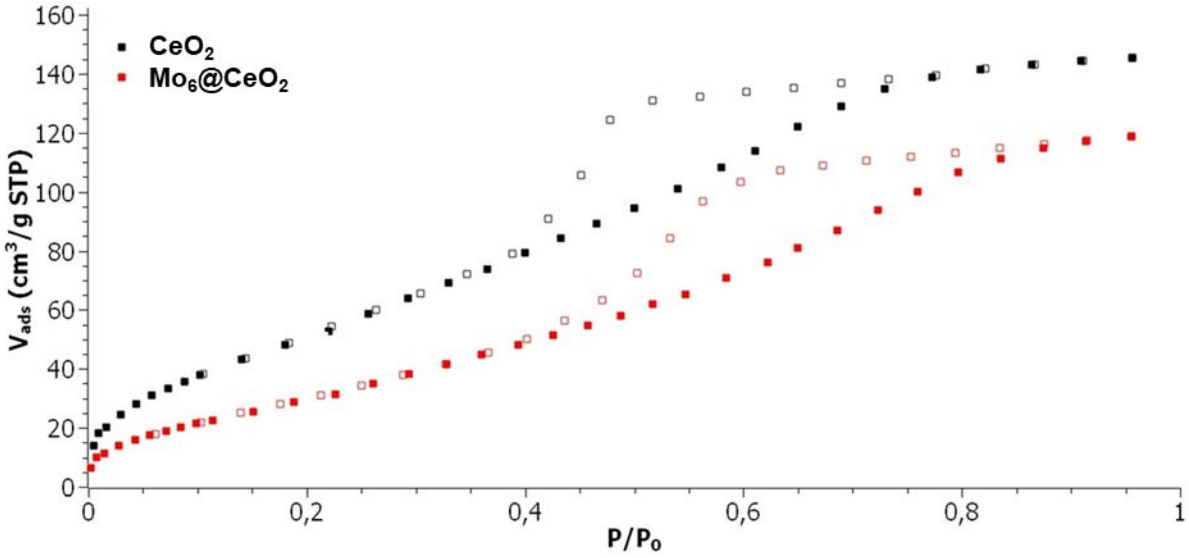

Figure S4. XPS spectra of the Mo3d, I3d and Ce3d core levels of **Mo_6**, **CeO_2** and **$Mo_6@CeO_2$** with corresponding fits (all characteristics are given in Table below).

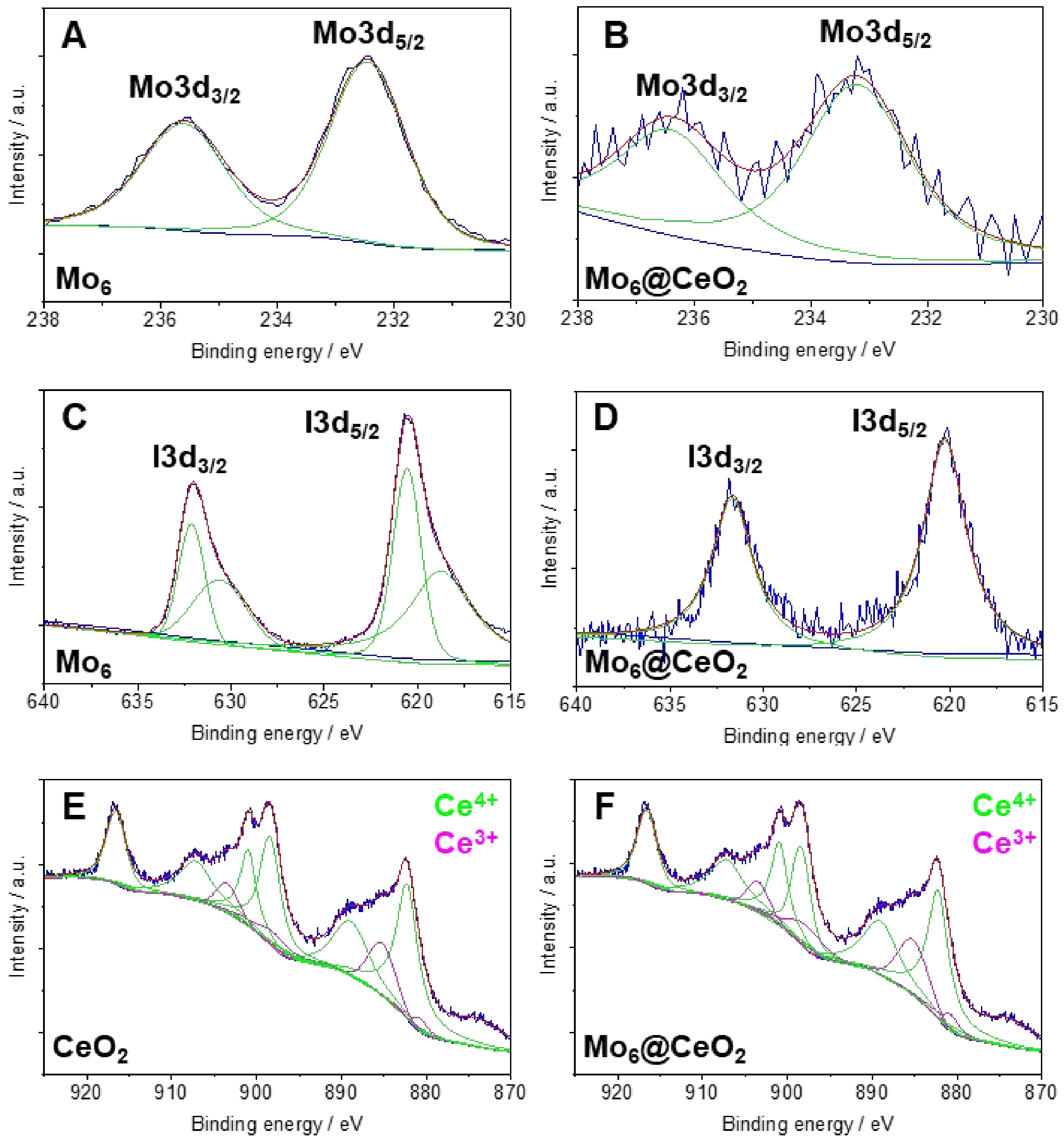

Sample	Core level	Center gravity	FWHM	AVG IntgP
**Mo_6**	$Mo3d_{5/2}$	232.5	1.6	60.3
	$Mo3d_{3/2}$	235.6	1.7	39.7
**$Mo_6@CeO_2$**	$Mo3d_{5/2}$	233.2	2.2	64.7
	$Mo3d_{3/2}$	236.4	2.2	35.3
**Mo_6**	$I3d_{5/2}$	620.6	1.8	66.1
	$I3d_{3/2}$	632.1	1.7	33.9
**$Mo_6@CeO_2$**	$I3d_{5/2}$	620.3	2.8	60.0
	$I3d_{3/2}$	631.7	2.7	40.0
**CeO_2**	$Ce3d_{5/2}$ (Ce^{3+})	880.7	2.2	1.7
	$Ce3d_{5/2}$ (Ce^{4+})	882.2	3.1	24.0
	$Ce3d_{5/2}$ (Ce^{3+})	885.2	3.7	7.8
	$Ce3d_{3/2}$ (Ce^{4+})	888.9	4.8	13.4
	$Ce3d_{5/2}$ (Ce^{3+})	897.9	4.1	3.7
	$Ce3d_{3/2}$ (Ce^{4+})	898.4	2.9	16.5
	$Ce3d_{3/2}$ (Ce^{4+})	901.0	2.3	9.9
	$Ce3d_{3/2}$ (Ce^{3+})	903.5	2.9	3.4
	$Ce3d_{3/2}$ (Ce^{4+})	907.1	4.6	10.0
	$Ce3d_{3/2}$ (Ce^{4+})	916.6	2.8	9.6
**$Mo_6@CeO_2$**	$Ce3d_{5/2}$ (Ce^{3+})	880.7	2.4	2.4
	$Ce3d_{5/2}$ (Ce^{4+})	882.2	3.0	22.0
	$Ce3d_{5/2}$ (Ce^{3+})	885.2	4.1	9.4
	$Ce3d_{3/2}$ (Ce^{4+})	889.0	4.9	14.2
	$Ce3d_{5/2}$ (Ce^{3+})	897.7	4.6	5.0
	$Ce3d_{3/2}$ (Ce^{4+})	898.4	2.6	12.3
	$Ce3d_{3/2}$ (Ce^{4+})	901.0	2.4	11.2
	$Ce3d_{3/2}$ (Ce^{3+})	903.5	3.0	3.5
	$Ce3d_{3/2}$ (Ce^{4+})	907.1	4.6	10.3
	$Ce3d_{3/2}$ (Ce^{4+})	916.6	2.8	9.7

Figure S5. Size distribution by number (**A**) and zeta potential distribution (**B**) of **Mo_6**, **CeO_2** and **Mo_6@CeO_2** in deionized water (pH~6), as obtained by dynamic light scattering. (**C**) Zeta potential distribution of **Mo_6**, **CeO_2** and **Mo_6@CeO_2** in methanol as obtained by dynamic light scattering.

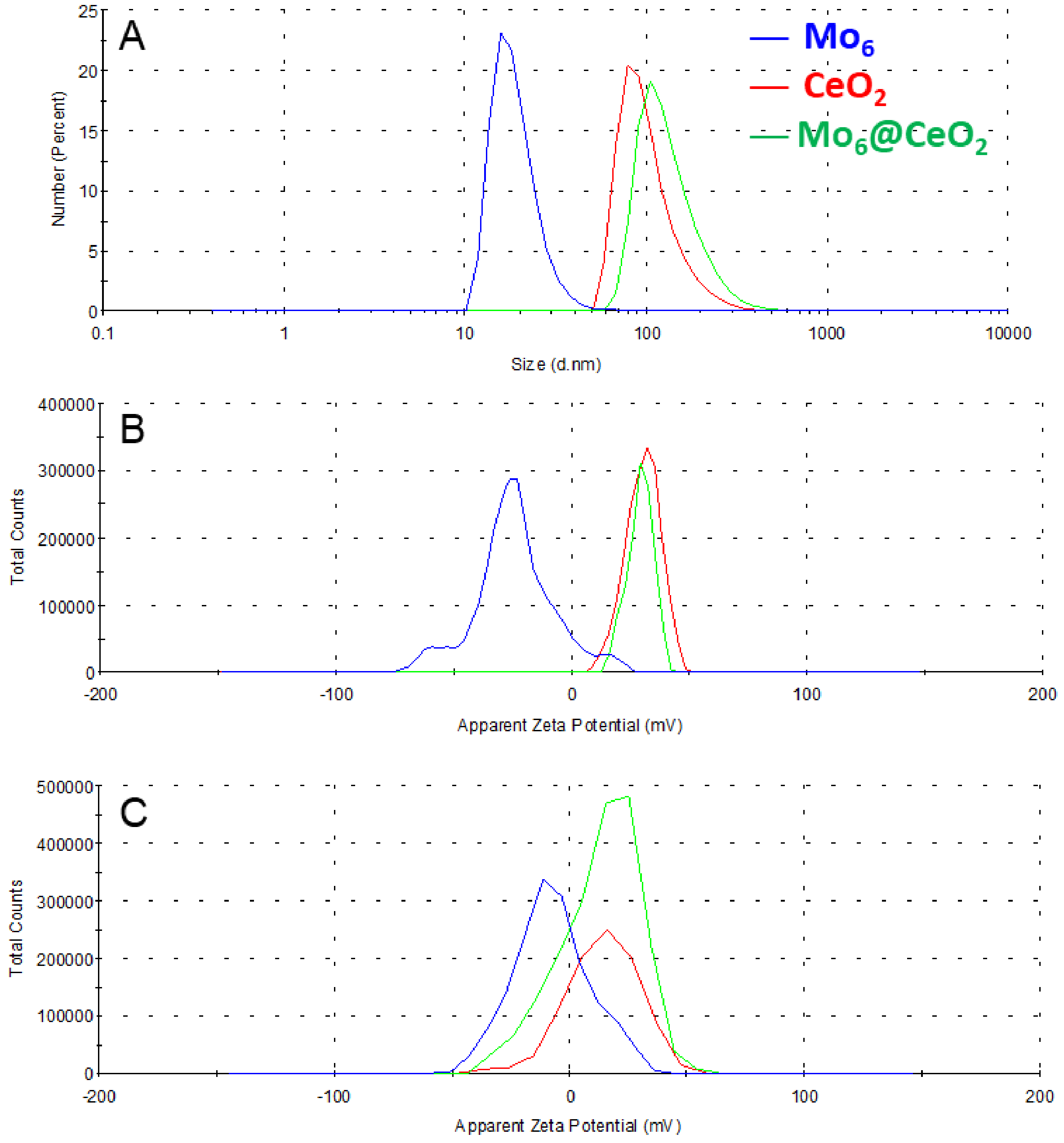

Figure S6. Phosphorescence decay kinetics of Ar-saturated (black) and air-saturated (red) aqueous dispersions of $\mathbf{Mo_6}$ and $\mathbf{Mo_6@CeO_2}$ excited at 400 nm and recorded at the maximum of emission.

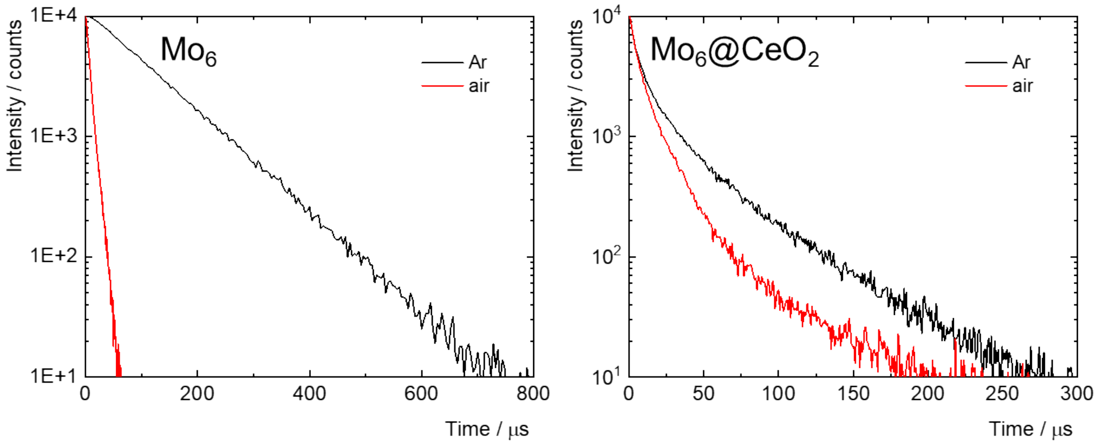

Kinetic analysis. Kinetic equation expressing change in concentration of BPS in time.

$C_t = C_0 e^{-kt}$ (S1),

where C_t is the concentration of BPS at time t (min), C_0 is the initial concentration of BPS, and k is the corresponding rate constant (min^{-1}). The rate constant (k) was determined by fitting experimental data to this equation.

The half-life ($\tau_{1/2}$, min) is defined as the time required for the concentration of BPS to decrease to half of its initial value. It was calculated using:

$\tau_{1/2} = \ln(2)/k$ (S2)

The degree of conversion (D, %) was calculated to quantify the extent of BPS adsorption/degradation after a given time:

$D = (1-C_t/C_0) \times 100$ (S3)

Table S1. Kinetic parameters of BPS adsorption in the dark and photocatalytic degradation under UV-A and solar light irradiation on **CeO_2** and **Mo_6@CeO_2**.[a]

Sample	In dark				Under UV-A			
	k_{dark}±SE /min^{-1}	$\tau_{1/2,dark}$ /min	R^2	D_{dark} (%)	k_{UV}±SE /min^{-1}	$\tau_{1/2,UV}$ /min	R^2	D_{UV} (%)
**CeO_2**	0.301 ±0.048	2.3	0.988	18.7	0.030 ±0.005	23.4	0.991	35.3
**Mo_6@CeO_2**	0.163 ±0.012	4.2	0.997	61.9	0.063 ‘ ±0.004	11.1	0.999	32.5

Sample	In the dark				Solar light irradiation			
	k_{dark}±SE /min^{-1}	$\tau_{1/2,dark}$ /min	R^2	D_{dark} (%)	k_{solar}±SE /min^{-1}	$\tau_{1/2,solar}$ /min	R^2	D_{solar} (%)
**CeO_2**	0.344 ±0.126	2.0	0.950	20.5	0.018 ±0.005	38.9	0.981	15.2
**Mo_6@CeO_2**	0.179 ±0.011	3.9	0.998	64.9	0.028 ±0.008	25.1	0.969	30.0

[a] k is the rate constant in the dark (k_{dark}), under UV-A (k_{UV}) and solar light (k_{solar}) irradiation (min^{-1}); τ is the half-life of the reaction in the dark ($\tau_{1/2,dark}$), under UV-A ($\tau_{1/2,UV}$) and solar light ($\tau_{1/2,solar}$) irradiation (min); D is the degree of conversion in dark (D_{dark}), under UV-A (D_{UV}) and solar light (D_{solar}) irradiation (%); SE is the standard error (min^{-1}); and R^2 is the goodness of fit of the model to the experimental data.

Figure S7. Kinetic curves of adsorption (in the dark) and UV-A photocatalytic degradation of phenol (initial concentration 10 mg mL[1]) on **Mo_6@CeO_2.** (2.5 mg mL^{-1})

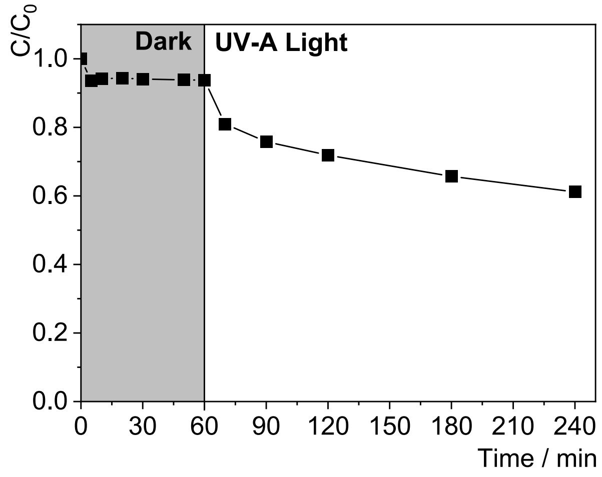

Figure S8. Chromatograms of the mobile phase (blank) and BPS standard (t_R = 4.82 min).

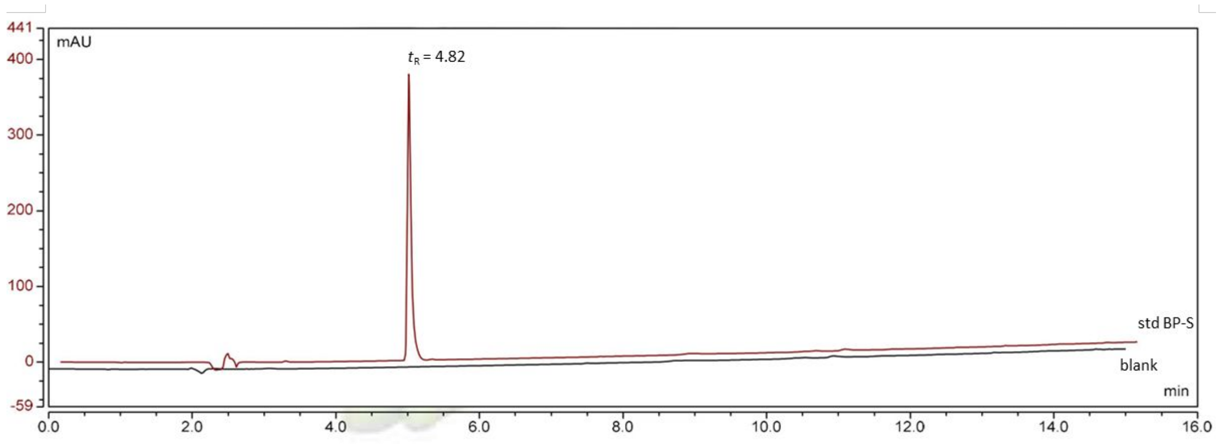

Figure S9. HRMS spectra of the BPS standard (t_R = 4.82 min).

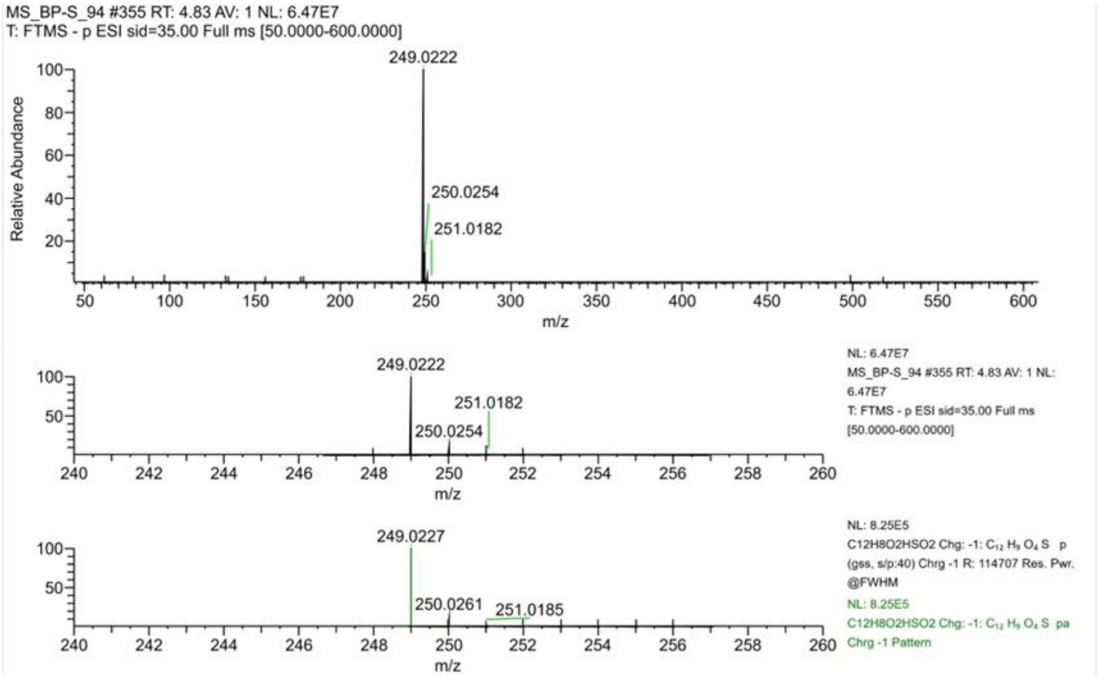

Figure S10. Time-resolved chromatograms of BPS products released to solution after BPS adsorption and photodegradation on **$Mo_6@CeO_2$** (blank, BPS standard and samples collected after 5, 10, 20, 30, 50, 60, 70, 90, 120, 180, and 240 min); 1 – HBSA (t_R = 1.79 min), 1` – HBSA iso (t_R = 2.01 min), 2 – $C_{12}H_{10}O_7S_2$ (t_R = 3.10 min), 3 – $C_6H_6O_7S_2$ (t_R = 3.58 min), 4 – phenol (t_R = 4.57 min), 5 - BPS (t_R = 4.82 min), 6 – BPS iso1 (t_R = 5.14 min), and 7 – BPS iso2 (t_R = 7.87 min).

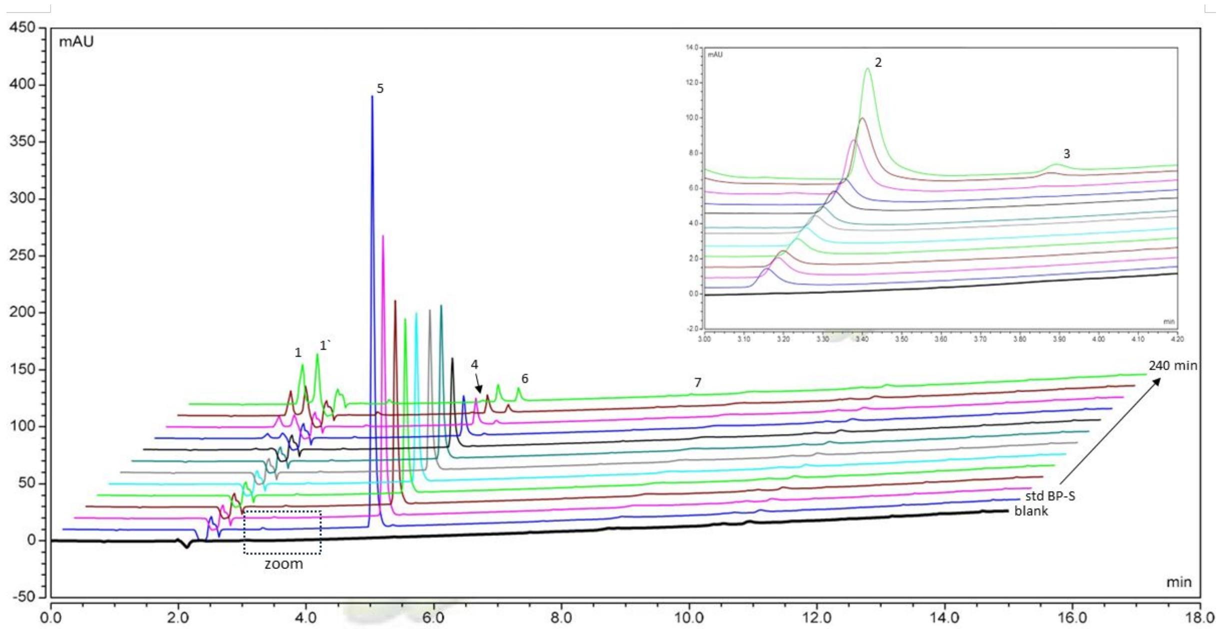

Figure S11. Time-resolved chromatograms of BPS products extracted from the surface of **$Mo_6@CeO_2$** (blank and extracts after 5, 60, 70, and 240 min); 1 – HBSA (t_R = 1.79 min), 1` – HBSA iso (t_R = 2.01 min), 2 – $C_{12}H_{10}O_7S_2$ (t_R = 3.10 min), 3 – $C_6H_6O_7S_2$ (t_R = 3.58 min), 4 – phenol (t_R = 4.57 min), 5 - BPS (t_R = 4.82 min), 6 – BPS iso1 (t_R = 5.14 min), and 7 – BPS iso2 (t_R = 7.87 min). Extraction was performed using ACN:H_2O (1:1, v/v).

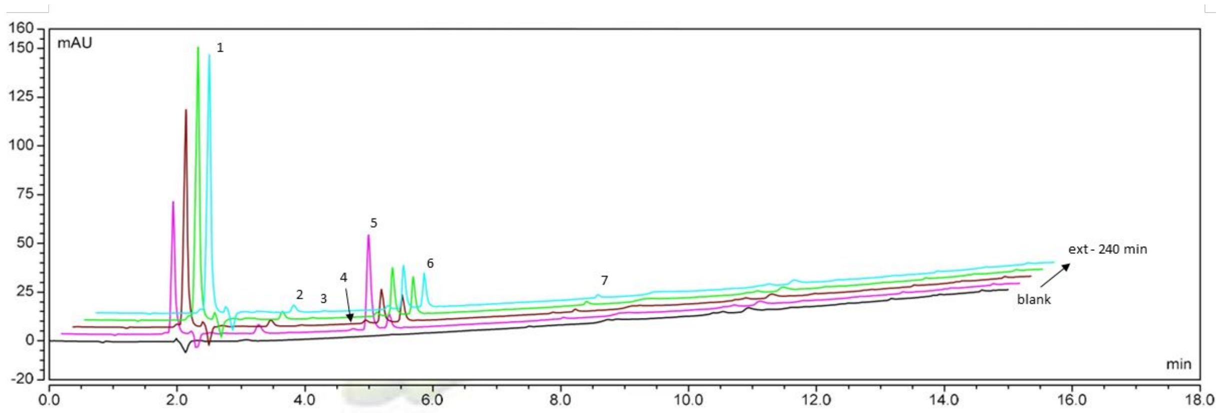

Figure S12. HRMS spectra of HBSA (t_R = 1.79 min).

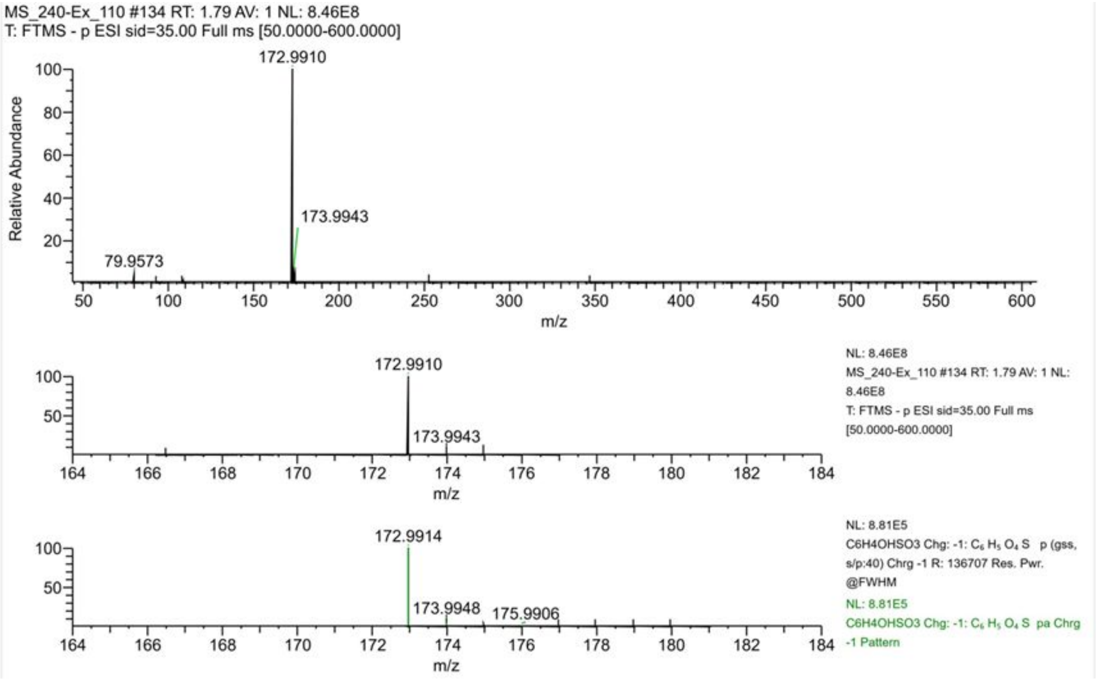

Figure S13. HRMS spectra of HBSA iso (peak 1'; t_R = 2.01 min) formed during photocatalytic degradation of BPS.

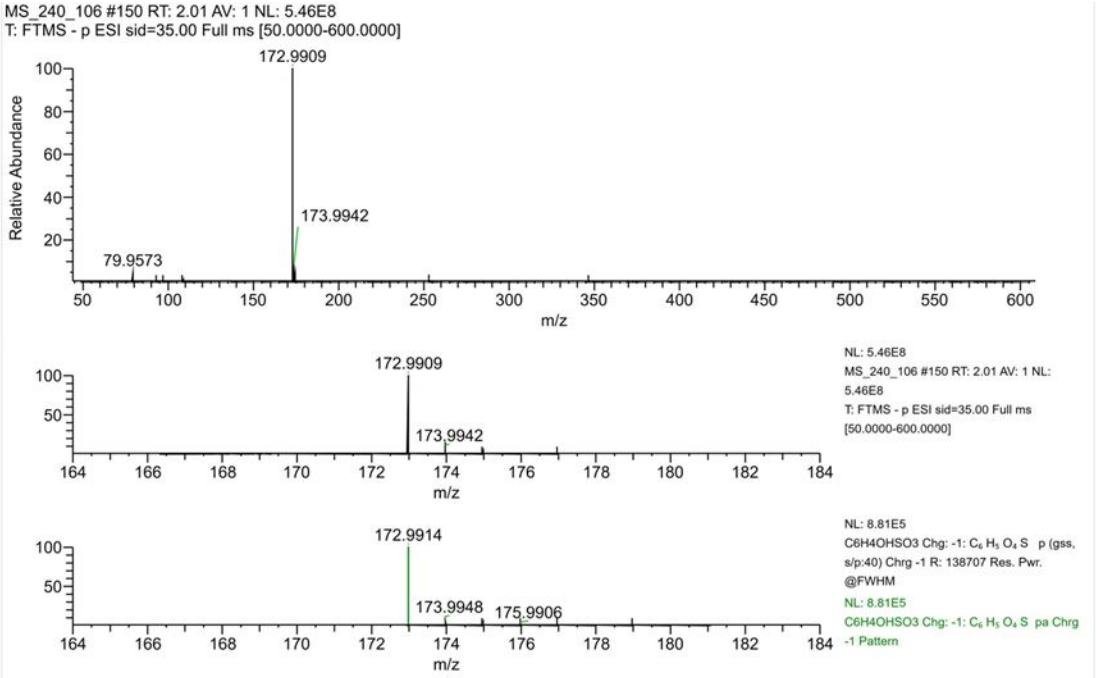

Figure S14. HRMS spectra of the peak 4 (phenol) (t_R = 4.57 min).

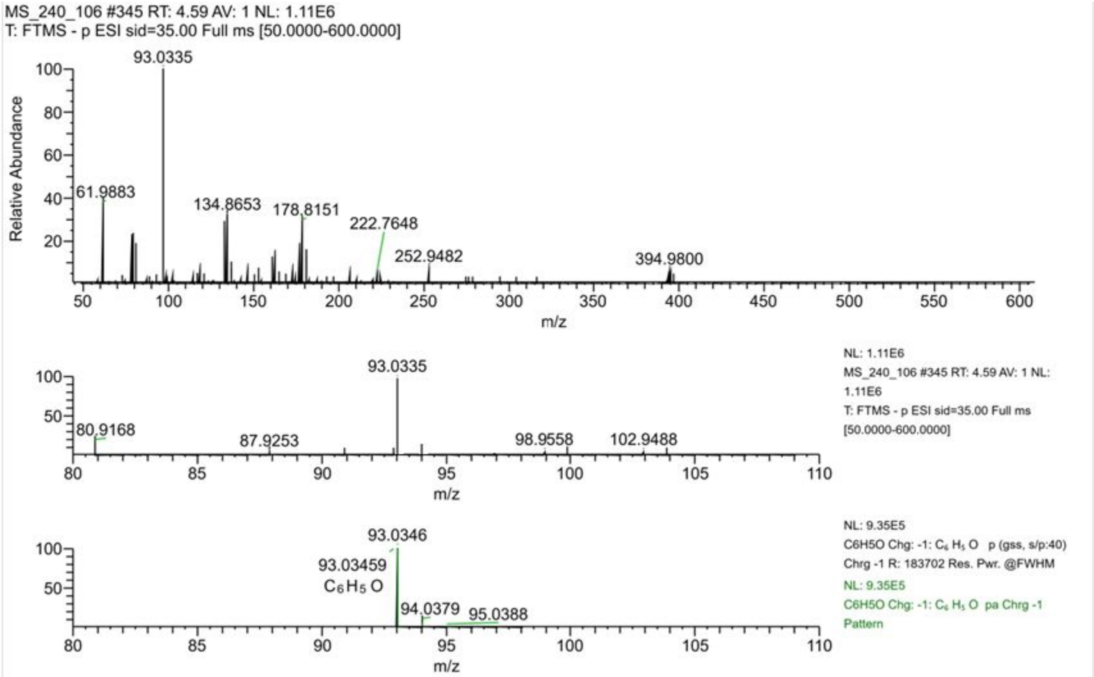

Figure S15. HRMS spectra of BPS (t_R = 4.82 min) and its isomers (BPS iso1 and BPS iso2) eluted at 5.14 (peak 6) and 7.87 min (peak 7).

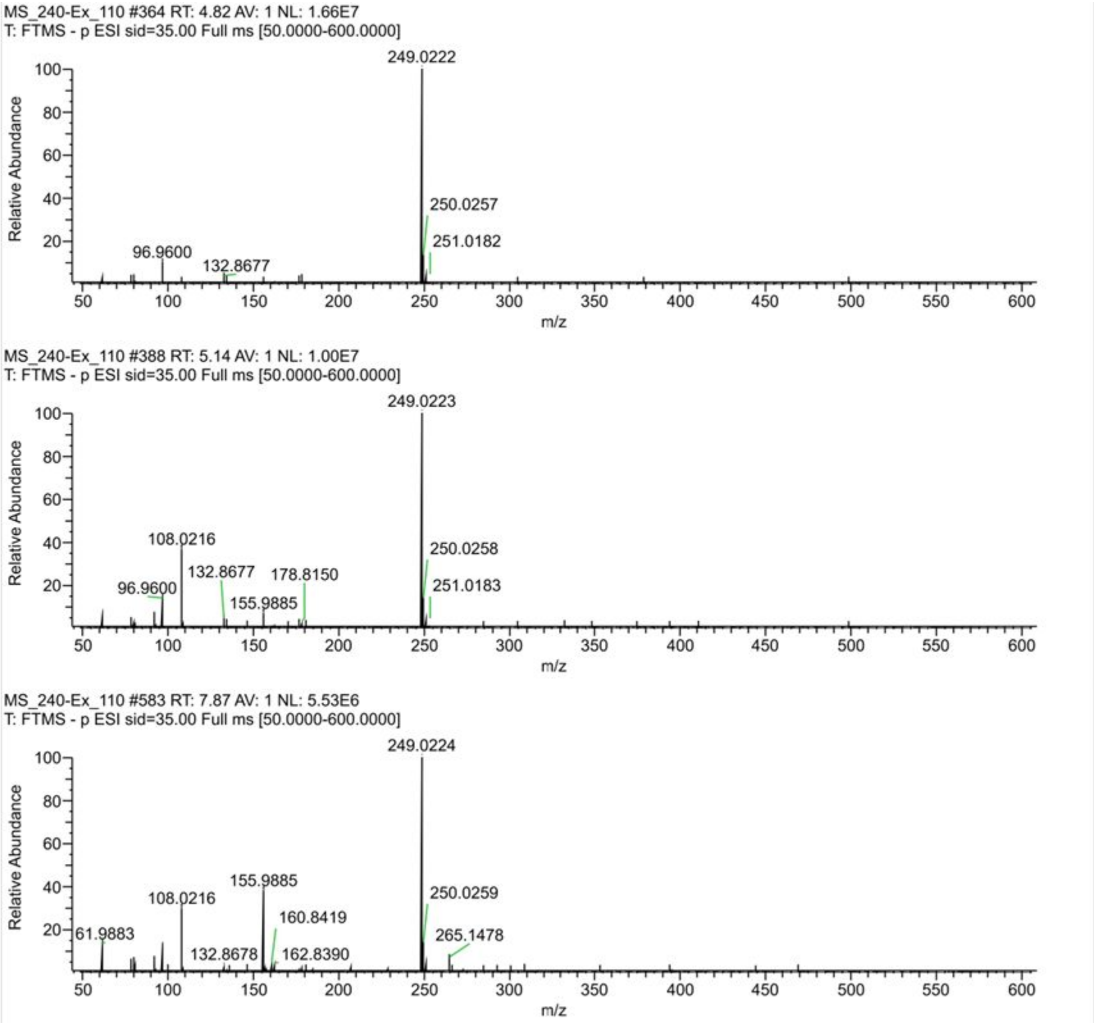

Figure S16. HRMS spectra of the peak 2 (t_R = 3.10 min).

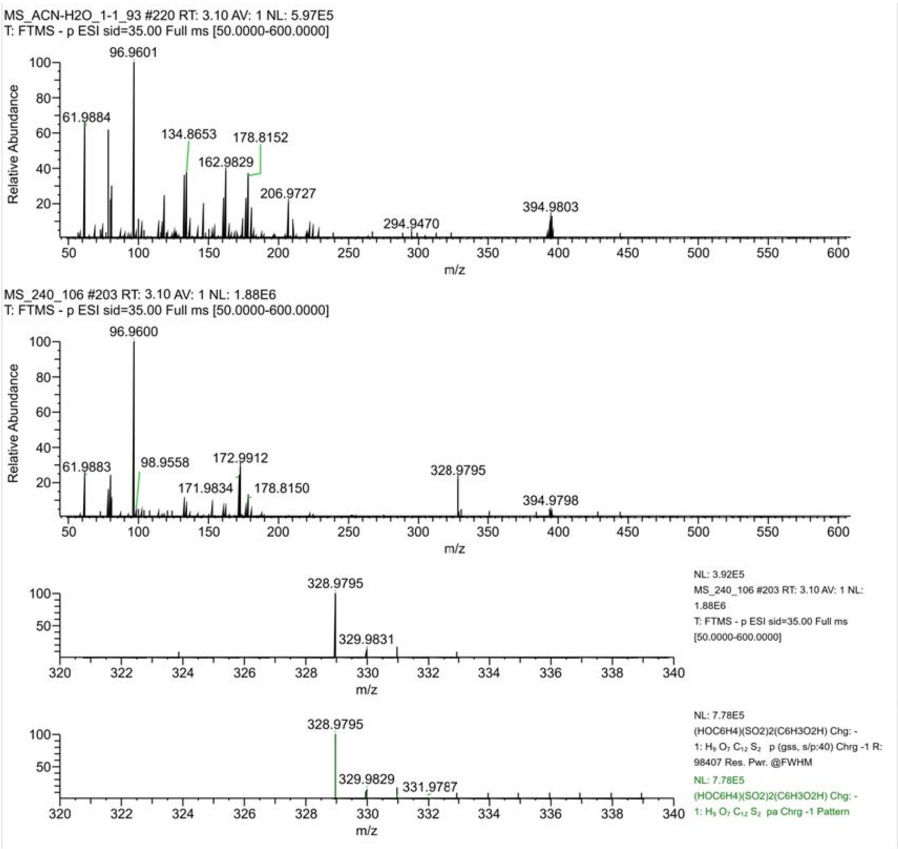

Figure S17. HRMS spectra of the peak 3 (t_R = 3.58 min).

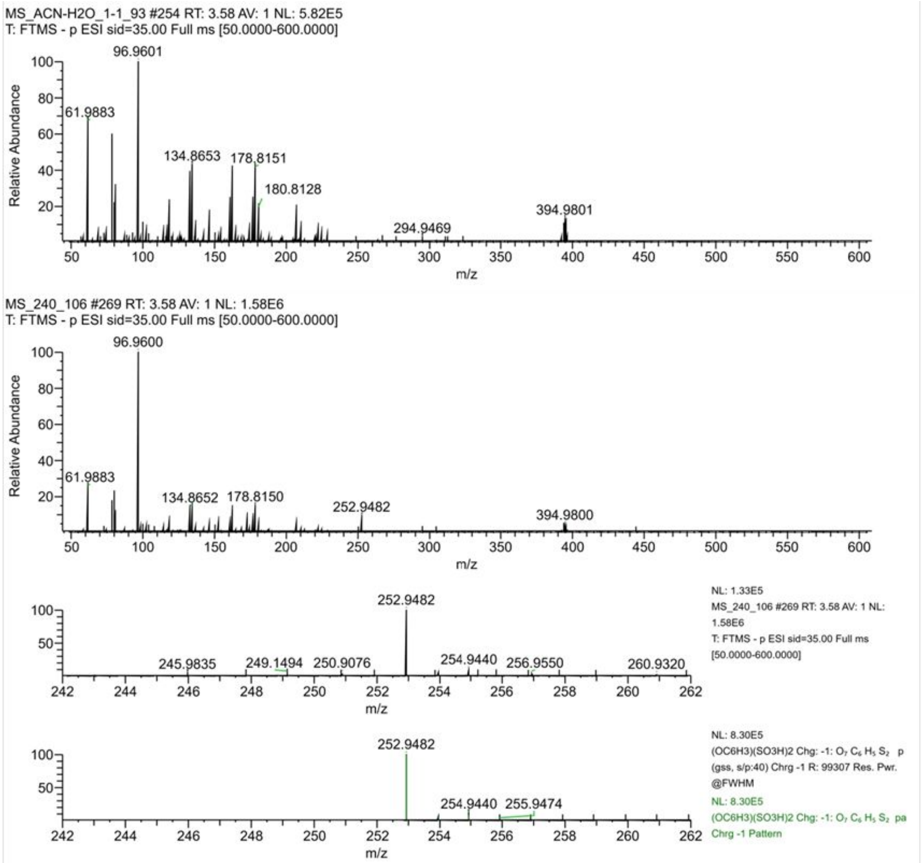

Figure S18. Time distribution of all products released into solution, resulting from BPS adsorption and UV-A photocatalytic degradation on **Mo_6@CeO_2**, expressed as the peak area from LC-HRMS analyses.

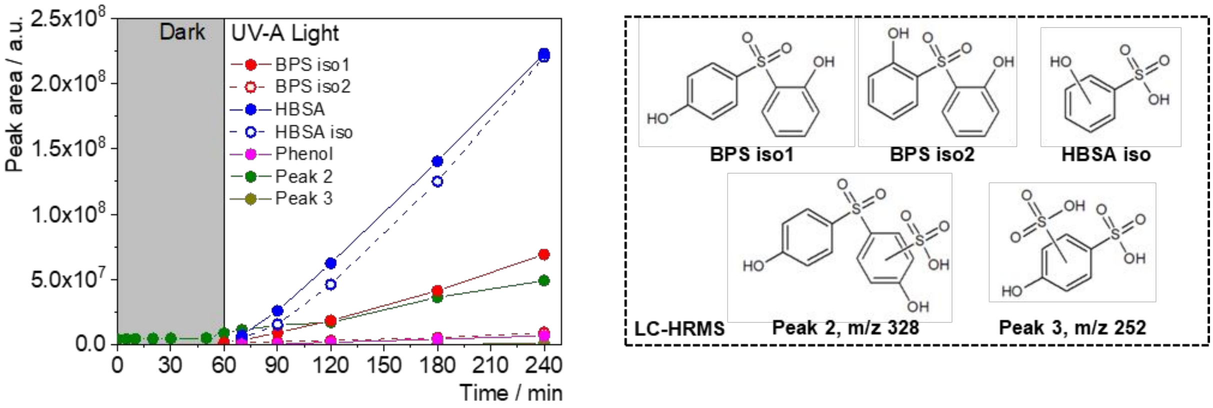

Supplement: Supplementary file 1 [file ic5c02157_si_001.pdf]
